# CRP deposition in human abdominal aortic aneurysm is associated with transcriptome alterations toward aneurysmal pathogenesis: insights from *in situ* spatial whole transcriptomic analysis

**DOI:** 10.3389/fimmu.2024.1475051

**Published:** 2024-12-16

**Authors:** Eun Na Kim, Hee Young Seok, Joon Seo Lim, Jiwon Koh, Jeong Mo Bae, Chong Jai Kim, Ga-Hyeon Ryu, You Jung Ok, Jae-Sung Choi, Chung-Hyun Cho, Se Jin Oh

**Affiliations:** ^1^ Department of Pathology, Seoul National University Hospital, Seoul National University College of Medicine, Seoul, Republic of Korea; ^2^ Department of Transdisciplinary Research and Collaboration, Genomics Core Facility, Seoul National University Hospital, Seoul, Republic of Korea; ^3^ Clinical Research Center, Asan Medical Center, University of Ulsan College of Medicine, Seoul, Republic of Korea; ^4^ Department of Pathology, Asan Medical Center, University of Ulsan College of Medicine, Seoul, Republic of Korea; ^5^ Genomics Core Facility, Biomedical Research Institute, Seoul National University Hospital, Seoul, Republic of Korea; ^6^ Department of Thoracic and Cardiovascular Surgery, Seoul Metropolitan Government-Seoul National University (SMG-SNU) Boramae Medical Center, Seoul National University College of Medicine, Seoul, Republic of Korea; ^7^ Department of Biomedical Sciences and Pharmacology , College of Medicine, Seoul National University, Seoul, Republic of Korea

**Keywords:** abdominal aortic aneurysm, C-reactive protein, spatial transcriptomics, STAT3, inflammation, apoptosis

## Abstract

**Background:**

We investigated the effects of C-reactive protein (CRP) deposition on the vessel walls in abdominal aortic aneurysm (AAA) by analyzing spatially resolved changes in gene expression. Our aim was to elucidate the pathways that contribute to disease progression.

**Methods:**

AAA specimens from surgically resected formalin-fixed paraffin-embedded tissues were categorized into the AAA–high CRP [serum CRP ≥ 0.1 mg/dL, diffuse and strong immunohistochemistry (IHC); n = 7 (12 cores)] and AAA–low-CRP [serum CRP < 0.1 mg/dL, weak IHC; n = 3 (5 cores)] groups. Normal aorta specimens obtained during heart transplantation were used as the control group [n = 3 (6 cores)]. Spatially resolved whole transcriptomic analysis was performed, focusing on CD68-positive macrophages, CD45-positive lymphocytes, and αSMA-positive vascular smooth muscle cells.

**Results:**

Spatial whole transcriptomic analysis revealed significant differential expression of 1,086, 1,629, and 1,281 genes between high-CRP and low-CRP groups within CD68-, CD45-, and αSMA-positive cells, respectively. Gene ontology (GO) analysis of CD68-positive macrophages identified clusters related to inflammation, apoptosis, and immune response, with signal transducer and activator of transcription 3 implicated across three processes. Notably, genes involved in blood vessel diameter maintenance were significantly downregulated in the high-CRP group. GO analysis of lymphocytes showed upregulation of leukocyte rolling and the apoptosis pathway, whereas, in smooth muscle cells, genes associated with Nuclear factor kappa B (NF-κB) signaling and c-Jun N-terminal Kinase (JNK) pathway were upregulated, and those related to blood pressure regulation were downregulated in the high-CRP group.

**Discussion:**

CRP deposition was associated with significant transcriptomic changes in macrophages, lymphocytes, and vascular smooth muscle cells in AAA, suggesting its potential role in promoting pro-inflammatory and apoptotic processes, as well as contributing to the degradation of vascular structure and elasticity.

## 1 Introduction

Abdominal aortic aneurysm (AAA) is an age-related disease characterized by an enlargement of the entire wall thickness of the aorta, with an arterial diameter exceeding 50% of the normal size (1). In the United States, approximately 200,000 new cases of AAA are diagnosed annually, and AAA-related deaths account for 2% of all deaths (2). AAA is often asymptomatic until rupture, at which point the fatality rate is over 50%. Key risk factors for AAA include advanced age, male sex, atherosclerosis, hypertension, and genetic predisposition. Particularly, age is closely related to the incidence of AAA, and the prevalence of AAA is increasing with the aging population (3).

The molecular mechanisms underlying the development of AAA remain largely unknown, and there are no effective drugs available to prevent or delay its progression (4). Recent studies have increasingly highlighted the crucial role of inflammation in the development and progression of AAA. Macrophages, T cells, B cells, and neutrophils infiltrate the AAA, secreting cytokines, chemokines, and proteases that create an inflammatory environment, which subsequently leads to the degradation of the extracellular matrix and weakening of the aortic wall (5, 6). Importantly, recent studies have revealed that the deposition of C-reactive protein (CRP) in the aorta contributes to the exacerbation of AAA. CRP is a well-known prognostic marker in various cardiovascular diseases including AAA; in patients with asymptomatic AAA, higher CRP levels are associated with faster aneurysm expansion (7) and increased short-term mortality (8). Additionally, higher CRP levels correlate with larger aneurysm diameters in patients with AAA (9).

Circulating serum CRP typically exists in a pentameric form and plays an anti-inflammatory role. However, when CRP encounters damaged cell membranes, it converts to a monomeric form, deposits in tissues, and exhibits strong pro-inflammatory characteristics (10, 11). Our research group has studied the association between CRP deposition and immune cell infiltration into the aortic walls of AAA (12, 13). Specifically, patients with high serum CRP levels not only had larger AAA diameters but also exhibited strong CRP deposition at the atherosclerosis-aortic wall interface and within the intima and media of resected aortic specimens (12). Furthermore, CRP deposition in these areas was colocalized with CD68+ macrophages and complement components, indicating a significant immune response. Proteomic profiling of the vascular wall from resected AAA tissues with high–CRP deposition and high–serum CRP levels revealed a dramatic shift toward a pro-inflammatory status, with enrichment in atherosclerotic and complement signaling pathways (12). Additionally, by utilizing CO-Detection by indEXing (CODEX) multiplexed tissue imaging of resected AAA, we found that AAAs with high serum CRP levels and strong tissue CRP deposition exhibited significant immune cell infiltration, particularly with the presence of M1-like macrophages (13). However, these observations have been limited to the protein level, and the underlying changes at the transcriptomic level have not been explored.

Therefore, in this study, we aimed to investigate the transcriptomic changes associated with CRP deposition in AAAs to elucidate the underlying mechanisms contributing to poorer prognosis and larger aortic diameter in patients with elevated CRP levels. We utilized *in situ* spatial-specific transcriptomic profiling with the GeoMx Digital Spatial Profiler to conduct whole transcriptome analysis in CRP-deposited AAA. This approach allowed us to elucidate the mechanisms by which mCRP contributes to AAA progression through detailed transcriptomic analysis.

## 2 Materials and methods

### 2.1 Patient selection

We used archived surgically resected specimens from patients who underwent elective surgery for AAA with a diameter of 4 cm or more. The aortic specimens were sectioned at 4-mm intervals for gross examination. Regions with atheromatous plaque deposition and thinned aortic walls were identified, and at least three sections were selected. These sections were stored in 10% buffered formalin for 1 day before being processed into formalin-fixed paraffin-embedded (FFPE) blocks. Slides were prepared from the FFPE blocks at a thickness of 4 µm. Hematoxylin and eosin staining, Masson trichrome special staining, and Elastic staining were performed to analyze the structural changes in the elastic lamellae and extracellular matrix of the aortic wall and to assess degeneration. Immunohistochemical staining was carried out using an anti-CRP antibody (rabbit polyclonal, ab32412, Abcam, Cambridge, UK), which detects both monomeric and pentameric forms of CRP, and an anti-mCRP antibody (mouse monoclonal, C1688, Sigma-Aldrich, Saint Louis, MO, USA), which selectively detects monomeric CRP.

Consistent with previous studies (12), patients with AAA with serum CRP levels of 0.1 mg/dL or higher and CRP immunohistochemical staining showing 3+ diffuse strong staining were classified as the high-CRP group. This group included 12 cores from seven patients. Patients with AAA with serum CRP levels below 0.1 mg/dL and CRP immunohistochemical staining showing weak, focal staining were classified as the low-CRP group. This group included five cores from three patients. Lastly, normal vascular tissues obtained during heart transplantation from three individuals were used as the normal control group, comprising six cores.

### 2.2 Ethical approval and consent to participate

The study protocol was approved by the Institutional Review Boards (IRBs) of Asan Medical Center (IRB number: S2020-0196) and SMG-SNU Boramae Medical Center (IRB number: 20-2022-111). The study protocol adhered to the relevant ethical guidelines and regulations.

Because the bioethical law in the Republic of Korea requires written informed consent when using biological specimens obtained since 2013, we conducted a transcriptomic analysis using Nanostring GeoMx on FFPE tissue blocks obtained in 2012 with anonymized patient information. Both IRBs approved the use of clinical data and the collection and utilization of biological samples for research purposes, exempting the need for formal written informed consent.

### 2.3 Tissue microarray preparation

FFPE AAA specimens were used to create tissue microarrays. In the high-CRP group, low-CRP group, and the control aorta group, 3-mm-sized punches were made to include atheroma, immune cell infiltration, and adjacent smooth muscle. One or two independent cores per patient were prepared, resulting in a total of 24 cores (10 cases in the experimental group and 3 cases in the control group). The tissue microarray was sectioned at a thickness of 4 µm, stained with hematoxylin and eosin, and digitized using the Pannoramic P250 Digital Slide Scanner (3D Histech).

### 2.4 Sample preparation for spatial transcriptomics

FFPE tissue samples were sectioned at a thickness of 4 µm using a microtome and mounted on positively charged slides (Leica Bond Plus slides, Cat# S21.2113.A). The sections were air-dried overnight at room temperature and stored at 4°C in a desiccator. FFPE sample preparation was performed according to the NanoString (Seattle, WA, USA) GeoMx Digital Spatial Profiling (DSP) Manual Slide Preparation User Manual (MAN 10150-02). Slides with mounted sections were baked for 3 h in a 60°C drying oven before paraffin removal. Paraffin was removed by immersing the slides in Citrisolv three times for 5 min each, followed by washes with 100% ethanol, 95% ethanol, and phosphate-buffered saline (PBS) solutions. Rehydration was performed in 1× Tris-EDTA (pH 9.0) at 100°C for 15 min, followed by a PBS wash. To expose the RNA target, sections were digested with Proteinase K (0.1 μg/mL) for 15 min at 37°C and then washed with PBS. Tissue morphology was preserved by incubating the sections in 10% neutral-buffered formalin (NBF) for 5 min, followed by washes with NBF Stop Buffer and 1× PBS. The human whole transcriptome atlas was hybridized to the sections overnight at 37°C. During hybridization, slides were covered with HybriSlip hybridization covers (Grace BioLabs, Bend, OR). After incubation, coverslips were removed by soaking in 2× saline/sodium citrate (SSC) buffer with 0.1% Tween-20. Two 25-min stringent washes were performed in 50% formamide in 2× SSC at 37°C to remove unbound probes, followed by additional washes in 2× SSC. For antibody morphology marker staining, samples were incubated in a blocking buffer (W buffer) for 30 min at room temperature in a humidity chamber. After 30 min, the W buffer was removed, and the sections were stained with the antibody morphology marker.

### 2.5 IF staining for morphology analysis and ROI/AOI selection

**Figure 1:**
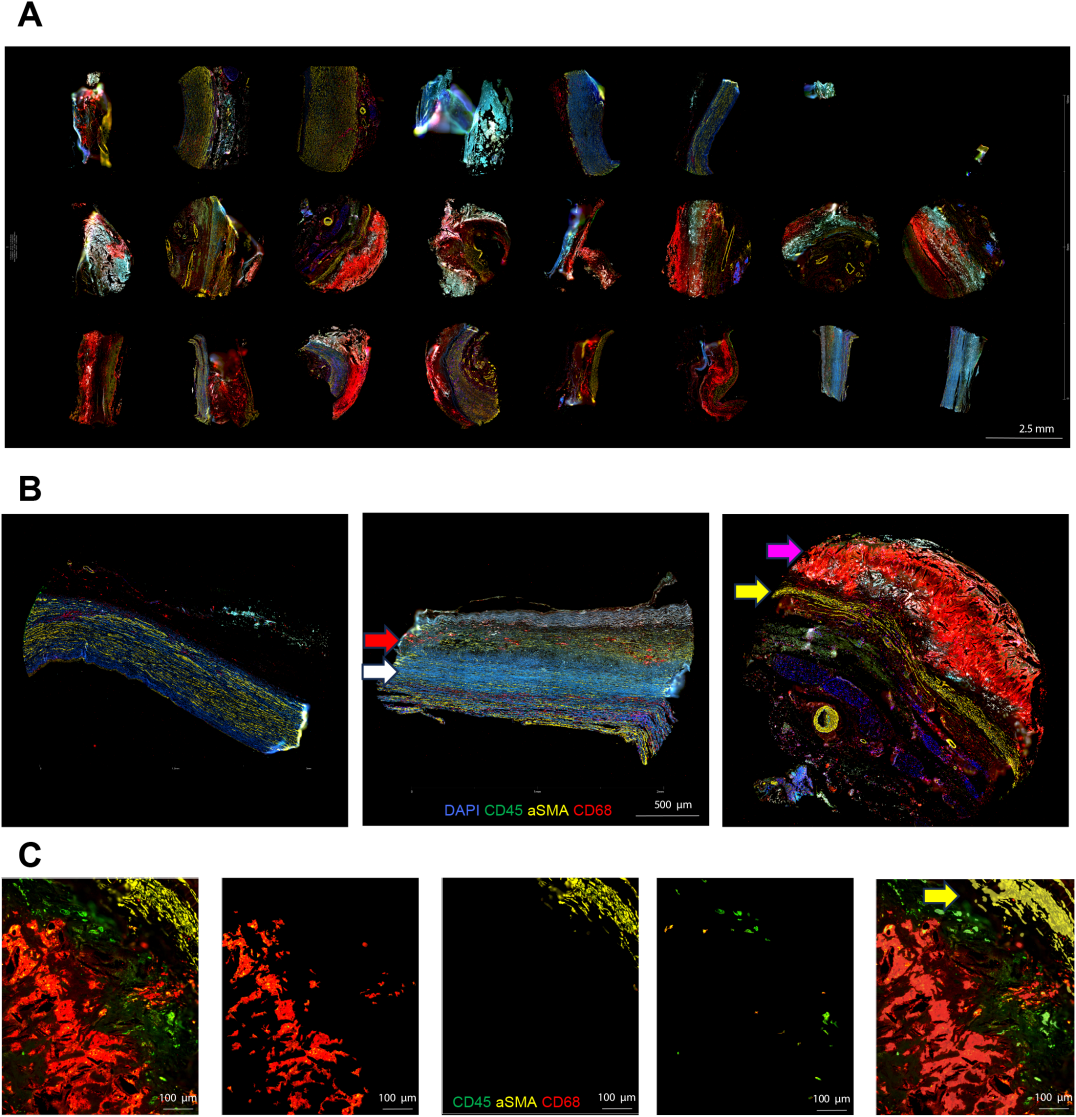
Morphologic marker staining and area of illumination selection in aortic tissue analysis. **(A)** Whole TMA slide scan view images for a normal aorta, AAA with low CRP, and AAA with high CRP. Each TMA section was prepared by creating 3-mm-diameter cores from the aortic wall, focusing on areas in contact with atheroma if present [AAA–low-CRP (n = 3), AAA–high-CRP (n = 7), and control group (n = 3)]. **(B)** Representative sections of a normal aorta, AAA–low CRP, and AAA–high CRP. The normal aorta primarily consists of αSMA-positive vascular smooth muscle cells with minimal inflammatory cell presence. In AAA–low CRP, the αSMA-positive vascular wall is thinner with fibrotic changes (white arrow), and CD68-positive macrophage infiltration is observed between fibrotic regions (red arrow). In AAA–high CRP, an extensively stained CD68-positive atherosclerotic plaque (magenta arrow) accumulates on a significantly thinned αSMA-positive vascular smooth muscle cell layer (yellow arrow). **(C)** Representative image of AOI segmentation using IF morphologic markers. The atheromatous plaque and vascular smooth muscle interface were stained simultaneously with CD45, αSMA, and CD68 markers. Vascular smooth muscle segmentation is highlighted (yellow arrow).

To perform transcriptomic analysis of specific cell types in aortic aneurysm, we used four fluorochromes (AF488, AF532, AF594, and AF647) to label the following immunofluorescence (IF) markers: αSMA (#ab202368, Abcam, Cambridge, UK), CD45 (#NBP2-34528AF532, Novus, CO, USA), and CD68 (#sc-20060AF647, Santa Cruz, TX, USA) (**Figure 1**). Sections were incubated with these markers for 1 h at room temperature. This staining allowed us to differentiate cell lineages and establish three areas of illumination (AOIs) per region of interest (ROI). Sections were then loaded onto the GeoMx Digital Spatial Profiler (NanoString, GMX-DSP). After fluorescence imaging, geometric ROIs were selected using circles and user-defined polygons by a cardiovascular specialist pathologist (ENK). ROIs were chosen to include high concentrations of CD45+ immune cells in close proximity to αSMA+ vascular smooth muscle walls and CD68+ atherosclerotic areas. ROIs were further categorized into AOIs on the basis of the expression of these IF markers for whole transcriptomic analysis.

### 2.6 Whole transcriptomic analysis

**Figure 2:**
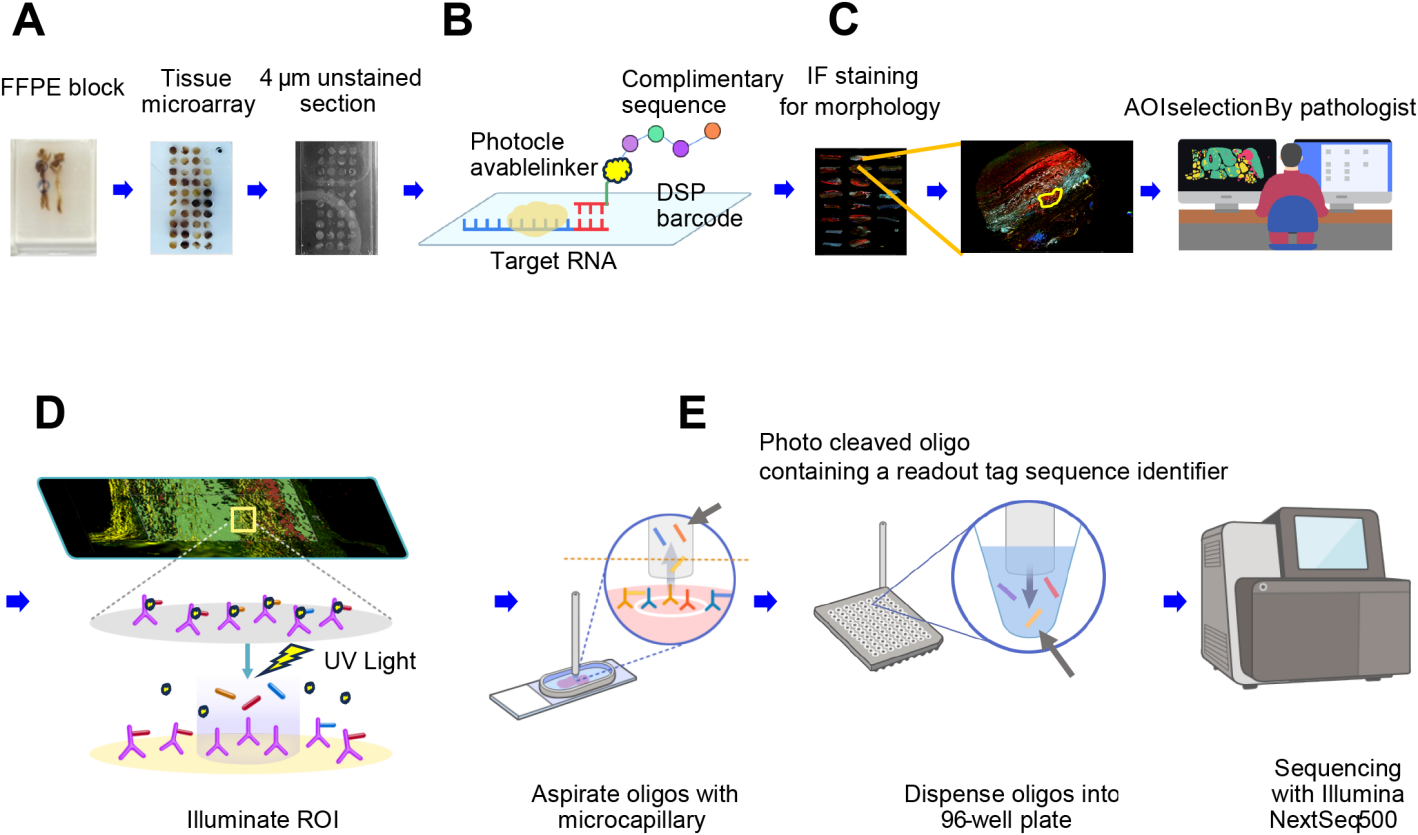
Schematic overview of the GeoMx platform for spatially resolved whole RNA transcriptome analysis. **(A)** Following the review of hematoxylin and eosin–stained slides, a pathologist selects regions of interest (ROIs) from the FFPE block of an aortic specimen. Tissue microarrays (TMAs) are created by punching 3-mm cores and sectioning them into micrometer-thin unstained sections. **(B)** A high-plex mixture of photocleavable oligo-linked probes and morphology-specific immunofluorescence (IF) antibodies is applied. **(C)** The pathologist uses IF morphologic markers to re-identify ROIs and defines the areas of illumination (AOIs). **(D)** The GeoMx instrument illuminates the AOIs with UV light, causing the photo-released oligos to detach and be deposited into a microtiter plate. **(E)** For NGS readout, the photo-cleaved oligos contain sequence identifiers that serve as readout tags. Each AOI is uniquely indexed during library preparation, pooled, and sequenced using the Illumina NextSeq 500 platform.

Spatially resolved whole transcriptomic expression profiling was performed using the NanoString GeoMx™ DSP RNA assay. The GeoMx platform for spatially resolved whole RNA transcriptome analysis is summarized in **Figure 2**. The Human NGS Whole Transcriptome Atlas RNA v1.0 probe kit, which consists of 18,677 RNA probes with ultraviolet (UV)–photocleavable indexing oligonucleotides, was used for this analysis. Each AOI was irradiated with UV light to induce photocleavage of the oligonucleotide probes conjugated to structural IF markers. The resulting photocleaved tags were released into a solution and collected in a 96-well plate. The collected aspirates contained photocleaved oligos, each tagged with a Readout Tag Sequence Identifier for identifying the biological target, and a Unique Molecular Identifier sequence for eliminating PCR duplicates in the subsequent data processing pipeline.

Additionally, the oligos contained Sequencing Primer Read 1 (SPR1) and Sequencing Primer Read 2 (SPR2) sequences for hybridization to GeoMx Seq Code primers during PCR amplification. The DSP aspirate was sealed with a permeable membrane and left to dry overnight at room temperature. Subsequently, the dried aspirate was reconstituted in 10 μL of diethylpyrocarbonate-treated water, and 4 μL was used for the PCR reaction. The PCR mixture comprised 2 μL of five PCR Master Mix, 4 μL of Seq Code primer, and 4 μL of the reconstituted aspirate. During PCR amplification, the GeoMx Seq Code primers introduced i5 and i7 indexing sequences (unique dual indexes) for multiplexing numerous GeoMx DSP aspirate sequences into a single sequencing run, along with P5 and P7 sequences for binding and amplification on Illumina flow cells. Following the PCR reaction, 1 μL of each PCR product was collected into a 1.5-mL tube and pooled. The pooled PCR product was quantified, and 1.2 times its weight in AMPure XP beads (Beckman Coulter, Indianapolis, IN) was added for purification. The purified libraries were sequenced on an Illumina NextSeq 500 with a minimum of 27 × 27 paired-end reads for the whole transcriptomic assay. The sequencing utilized paired-end 75 cycles.

### 2.7 Data preprocessing

To obtain clean data, preprocessing was performed using the GeoMx^®^ DSP Analysis Suite (GEOMX-0181) version 2.3.0.268. A quality check was conducted on the GeoMx DSP raw data. Initially, raw sequencing FASTQ files were compiled with the configuration file specifying the processing parameters. Adapter sequences were computationally removed to produce trimmed reads, and overlapping paired-end reads were merged to generate stitched reads. These stitched reads were then aligned to the RTS-ID barcodes in the reference assay, creating aligned reads and assigning raw counts to biological target names. Unique molecular identifiers (UMIs) were used to remove PCR duplicates, resulting in deduplicated reads. Technical signal quality control (QC) involved verifying that the raw read threshold exceeded 1,000 raw reads, the percentage of aligned reads surpassed 80% of the total raw reads, and sequencing saturation was greater than 50%. For technical background QC, we ensured that the negative probe count geomean, indicating the level of technical noise, was above 4, and the No Template Control count, which is used to detect contamination in the library preparation, was below 1,000. Additionally, spatial profiling parameters were checked to confirm a minimum nuclei count above 200 and a minimum surface area exceeding 7,000 µm².

### 2.8 Bioinformatics and statistical analysis: pathway and system biology analysis

To delineate the molecular mechanisms underlying the aggravation of AAA with CRP deposition, we performed pathway enrichment and systemic biological analyses based on whole transcriptomic expression levels through similar approaches as previously reported (14). Differentially expressed gene (DEG) analysis was conducted by comparing transcriptomic profiles between the high-CRP and low-CRP groups for CD68-positive macrophages, CD45-positive lymphocytes, and αSMA-positive smooth muscle cells of the aortic wall. To identify DEGs, we utilized the DESeq2 package in R (15) (version 3.6.2). We compared the differences in gene expression levels according to the level of CRP deposition in AAA, between the high-CRP and low-CRP groups. Genes with a false discovery rate < 0.1 by the Benjamini–Hochberg correction were considered as DEGs. The resulting gene lists underwent pathway enrichment analysis to determine the biological pathways most affected by CRP deposition. For the analyses of Gene Ontology (GO), we used DAVID (16) and REVIGO (http://revigo.irb.hr), which clusters semantically similar terms and selects representative terms, streamlining the list and providing a concise overview of enriched pathways (17). We employed STRING (Search Tool for the Retrieval of Interacting Genes/Proteins) (18) to construct protein–protein interaction networks (PPI). This analysis identified key regulatory transcriptomes and their interactions, clustering proteins with similar functions and highlighting significant interaction patterns.

### 2.9 Validation using immunohistochemistry

IHC validation for Phospho-Stat3 (signal transducer and activator of transcription 3, rabbit monoclonal IgG, Tyr705, D3A7, #9145, Cell Signaling Technology) was performed to confirm the translocation of phosphorylated STAT3 to the nucleus. For the secondary antibody, the OptiView DAB IHC Detection Kit (Ventana Medical Systems) was used. IHC was carried out on the Leica BOND Rx^®^ using the OptiView Universal DAB Detection Kit (Ventana). The IHC slides were digitized, and positive staining in the nuclear localization of the atheroma’s intima was quantified using QuPath software (19). The mean optical density of DAB staining in the nucleus was compared between the high-CRP and low-CRP groups, and statistical significance was determined using the Mann–Whitney–Wilcoxon test two-sided with Bonferroni correction.

## 3 Results

### 3.1 Characteristics of the study patients

The patient cohort in this study is identical to that of a previous study on AAA utilizing CODEX multiplexed tissue imaging. Therefore, the patient characteristics are the same as those reported in the previous studies (12, 13). The maximal diameter of aortic aneurysms was 6.3 ± 1.5 cm in the high-CRP group and 5.8 ± 0.3 cm in the low-CRP group. Additional information can be found in our previous study (12). The high-CRP group (serum CRP ≥ 0.1 mg/dL, diffuse and strong IHC) included seven patients (12 cores), whereas the low-CRP group (serum CRP < 0.1 mg/dL, weak IHC) included three patients (five cores). The control group included normal aorta specimens obtained during heart transplantation from three patients (six cores).

### 3.2 Spatial transcriptomic profiling results

Using morphologic markers CD45, CD68, and αSMA, we performed segmentation on the 23 cores, resulting in a total of 72 AOIs for analysis. All AOIs passed QC. We conducted whole transcriptomic analysis separately for CD68-positive macrophages, CD45-positive lymphocytes, and αSMA-positive smooth muscle cells, and their respective results are outlined below.

### 3.3 CD68-positive macrophages

#### 3.3.1 DEGs according to the level of CRP deposition

**Figure 3:**
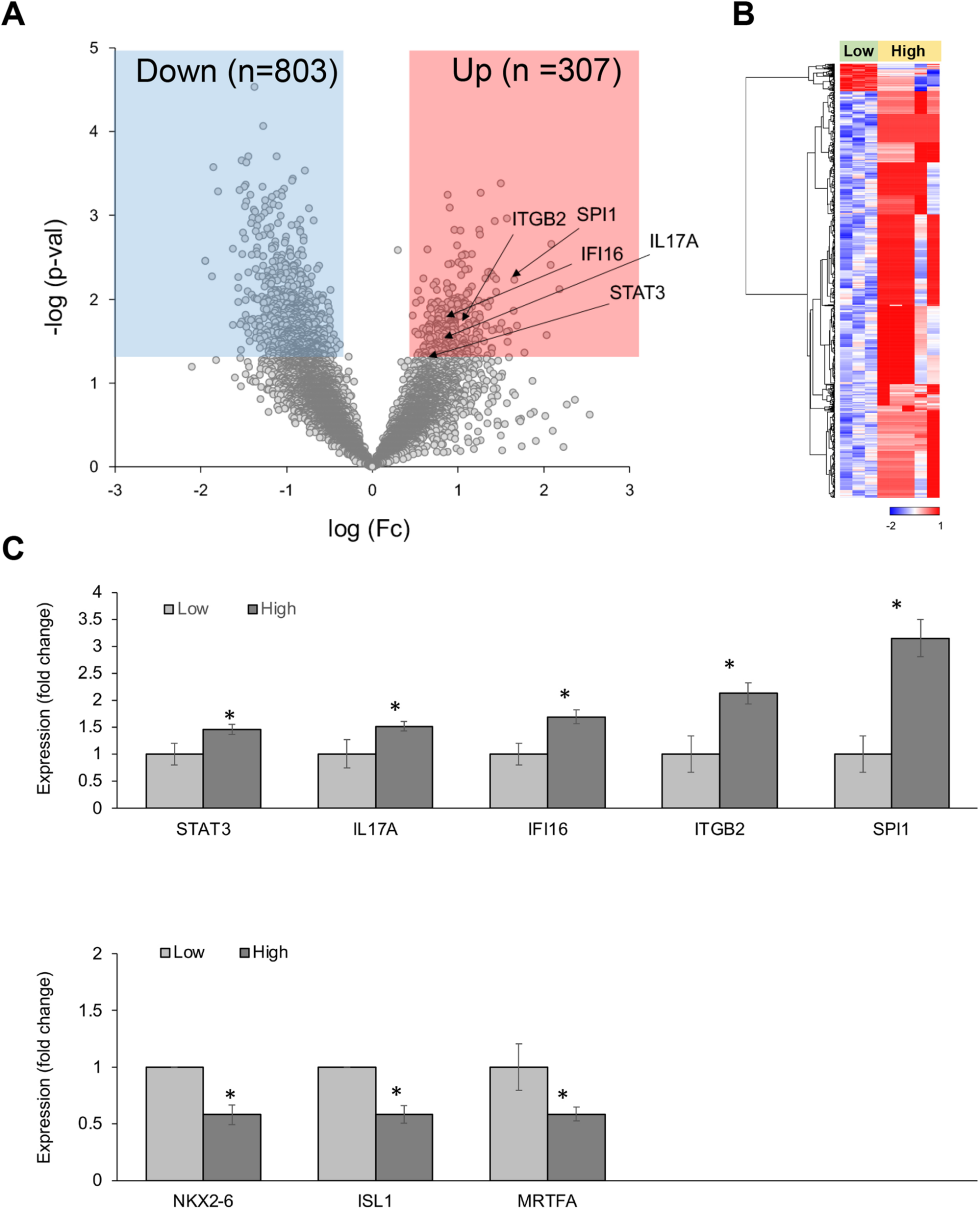
Differentially expressed genes (DEGs) in CD68-positive macrophages from spatial whole transcriptomic analysis [AAA–high CRP (n = 7) versus AAA–low CRP (n = 3)]. **(A)** Volcano plot displaying highly expressed DEGs in CD68-positive macrophages based on the degree of CRP deposition in AAA, using log_10_ fold changes. **(B)** Clustered heatmap of the selected ROIs showing whole transcriptomic expression fold changes (average gene expression, z-score) in relation to CRP levels in AAA. Approximately 1,100 genes were differentially expressed according to the CRP deposition level. **(C)** Key DEGs between the high-CRP and low-CRP groups from **(A)**. The asterisk symbol (*) indicates statistical significance at p < 0.05.

In CD68-positive macrophages, 1,110 DEGs were observed between the high-CRP and low-CRP groups (**Figure 3**). Using Student’s *t*-test supported by a p-value under 0.05, we identified 803 downregulated DEGs and 307 upregulated DEGs in the high-CRP group compared to those in the low-CRP group. As shown in the heatmap (**Figure 3B**), macrophage gene expression clusters distinctly according to CRP levels.

Among 307 upregulated genes, five genes—*STAT3, IL-17A, IFI16, ITGB2*, and *SPI1*—were notably upregulated in the high-CRP group. Among 803 downregulated genes, *MRTFA* (myocardin-related transcription factor A), which is involved in the survival and proliferation of pro-atherogenic macrophages (20) and plays a protective role in the vascular wall by mitigating vascular degeneration (21), was significantly downregulated in CD68-positive macrophage in AAA–high-CRP group (**Figure 3C**).

#### 3.3.2 Gene ontology analysis

**Figure 4:**
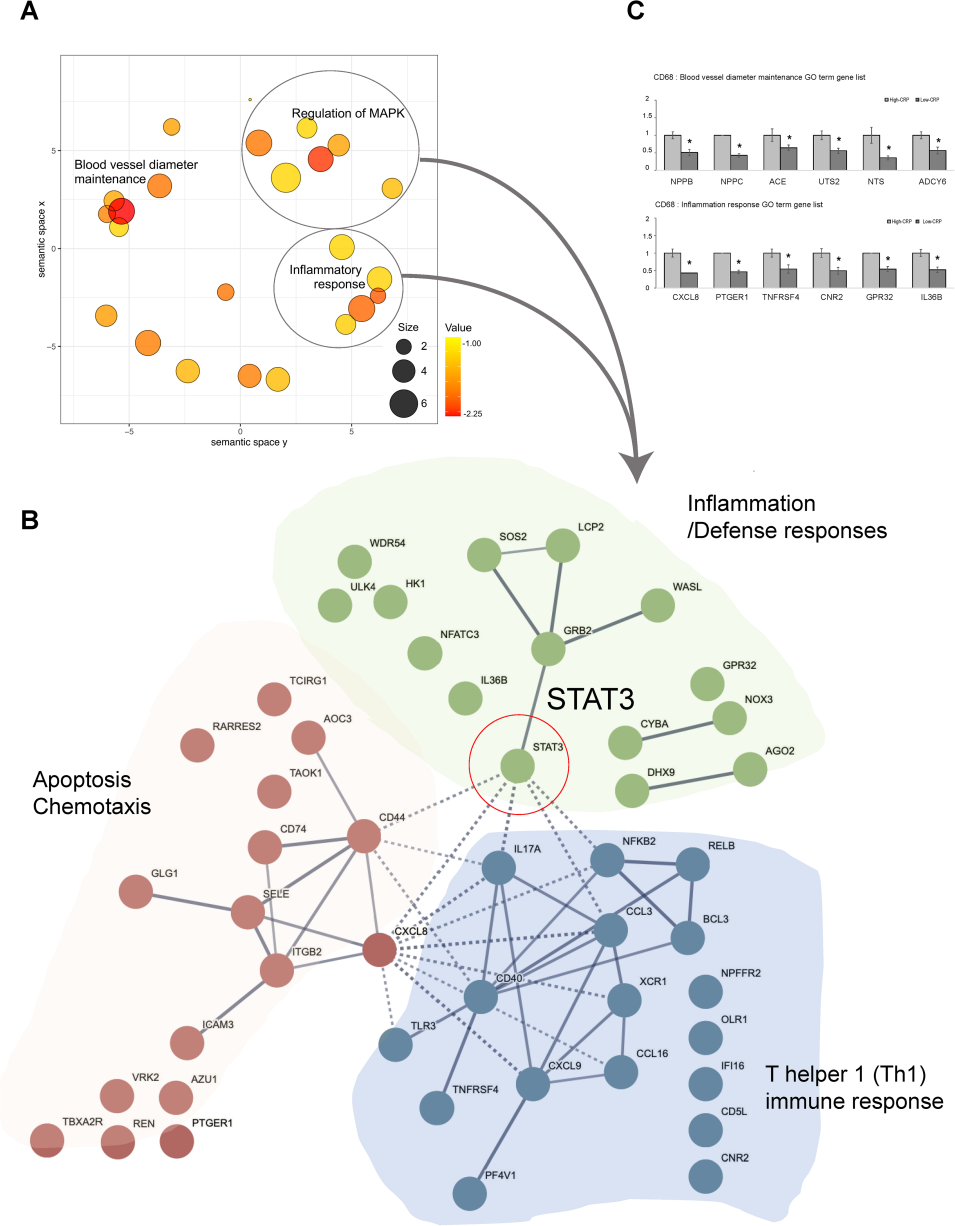
Functional enrichment and protein–protein interaction analysis of GO terms related to CRP deposition in AAA tissues: in CD68-positive macrophages [AAA–high CRP (n = 7) versus AAA–low CRP (n = 3)]. **(A)** Scatter plot from REVIGO illustrating cluster representatives after redundancy reduction in a two-dimensional space. Multidimensional scaling was applied to a matrix of the 34 GO terms’ semantic similarities. Notable GO terms identified include inflammatory response and blood vessel diameter maintenance. The bubble size indicates the frequency of the GO terms in the underlying GOA database, with color shading representing user-supplied p-values (legend in the lower right corner). **(B)** Potential enriched GO pathways in CD68+/CRP (high/low) regions. A total of 34 statistically significant biological process GO terms were observed. Further analysis highlighted representative GO terms such as inflammatory responses and MAPK cascade. Protein–protein interaction analysis using the STRING program with high confidence revealed that “*STAT3*” connects two other clusters associated with apoptosis and immune responses within the inflammation cluster. **(C)** GO analysis results of genes associated with “blood vessel diameter maintenance,” and “inflammation response.” DEG, differentially expressed gene; Down, downregulated; Up, upregulated; AAA–high CRP, abdominal aortic aneurysm with high CRP; AAA–low CRP, abdominal aortic aneurysm with low CRP. The asterisk symbol (*) indicates statistical significance at p < 0.05.

GO analysis of DEGs between the high-CRP and low-CRP groups in CD68-positive macrophages revealed a significant enrichment of 34 biological processes. Using REVIGO (17) analysis to reduce redundancy, we applied multidimensional scaling to the matrix of the 34 GO terms’ semantic similarities and projected them into a two-dimensional space. In this two-dimensional representation, “blood vessel diameter maintenance,” “inflammatory response,” and “regulation of MAP kinase cascade” emerged as representative terms (**Figure 4A**).

To investigate the physical and functional associations of these pathways, we utilized the PPI analysis tool, STRING. We found that *STAT3* links two other clusters associated with apoptosis and immune responses within the inflammation cluster (**Figure 4B**), suggesting that STAT3 plays a central role in connecting these three clusters both biologically and functionally. Furthermore, we examined the genes that were either upregulated or downregulated in the high-CRP group according to each significantly identified GO term.

In terms of the genes related to the GO term “Inflammatory Response,” the high CRP group showed significant decreases in *CXCL8, PTGER1, TNFRSF4, CNR2, GPR32, *and *IL-36B* (**Figure 4C**). CXCL8 promotes M2 polarization (22); PTGER1 (prostaglandin E2 receptor 1) fine-tunes the inflammatory response and M1-M2 macrophage polarization; CNR2 [cannabinoid receptor 2 (23, 24)] facilitates the polarization from the pro-inflammatory M1 phenotype to the anti-inflammatory M2 phenotype and inhibits the NF-κB pathway to mitigate inflammation; GPR32 (G protein–coupled receptor 32) resolves inflammation and reduces atherosclerosis (25, 26); and IL-36B (interleukin-36 beta) promotes phagocytosis and clearance in macrophages (27). The downregulation of genes related to M2 polarization and anti-inflammation in the high-CRP group is consistent with previous studies, indicating that CRP deposition creates a pro-inflammatory environment and M1 polarization (13).

In terms of genes corresponding to the GO term “blood vessel diameter maintenance,” *NPPB, NPPC, ACE, UTS2, NTS, *and *ADCY6* were downregulated in the CD68-positive macrophages in the high-CRP group (**Figure 4C**). These protective role genes include *NPPB, NPPC, ACE, UTS2, NTS,* and *ADCY6*. NPPB (natriuretic peptide B) plays a role in protecting endothelial function (28, 29). Similarly, NPPC (natriuretic peptide precursor C) ensures vascular elasticity and expansion, thereby maintaining vascular health and protecting against hypertension-related damage (30). ACE (angiotensin-converting enzyme) expressed in macrophages contributes to lipid handling capacity (31). UTS2 (urotensin2) (32) maintains vascular integrity, regulates blood pressure, and preserves the structural and functional integrity, as well as the elasticity and function, of blood vessels. NTS2 (neurotensin receptor 2) regulates endothelial and smooth muscle function (33). Additionally, ADCY6 (adenylate cyclase type 6) facilitates the release of vasoactive substances like nitric oxide (NO) through ADCY6-mediated cAMP production in macrophages, leading to VSMC relaxation and vasodilation (34). The decrease in the expression of these genes responsible for maintaining vessel diameter, vascular tone, and structural integrity in the high-CRP group suggests that CRP deposition is closely associated with the compromised vascular integrity observed in aortic aneurysms.

### 3.4 CD45-positive lymphocytes

#### 3.4.1 DEGs according to the level of CRP deposition

**Figure 5:**
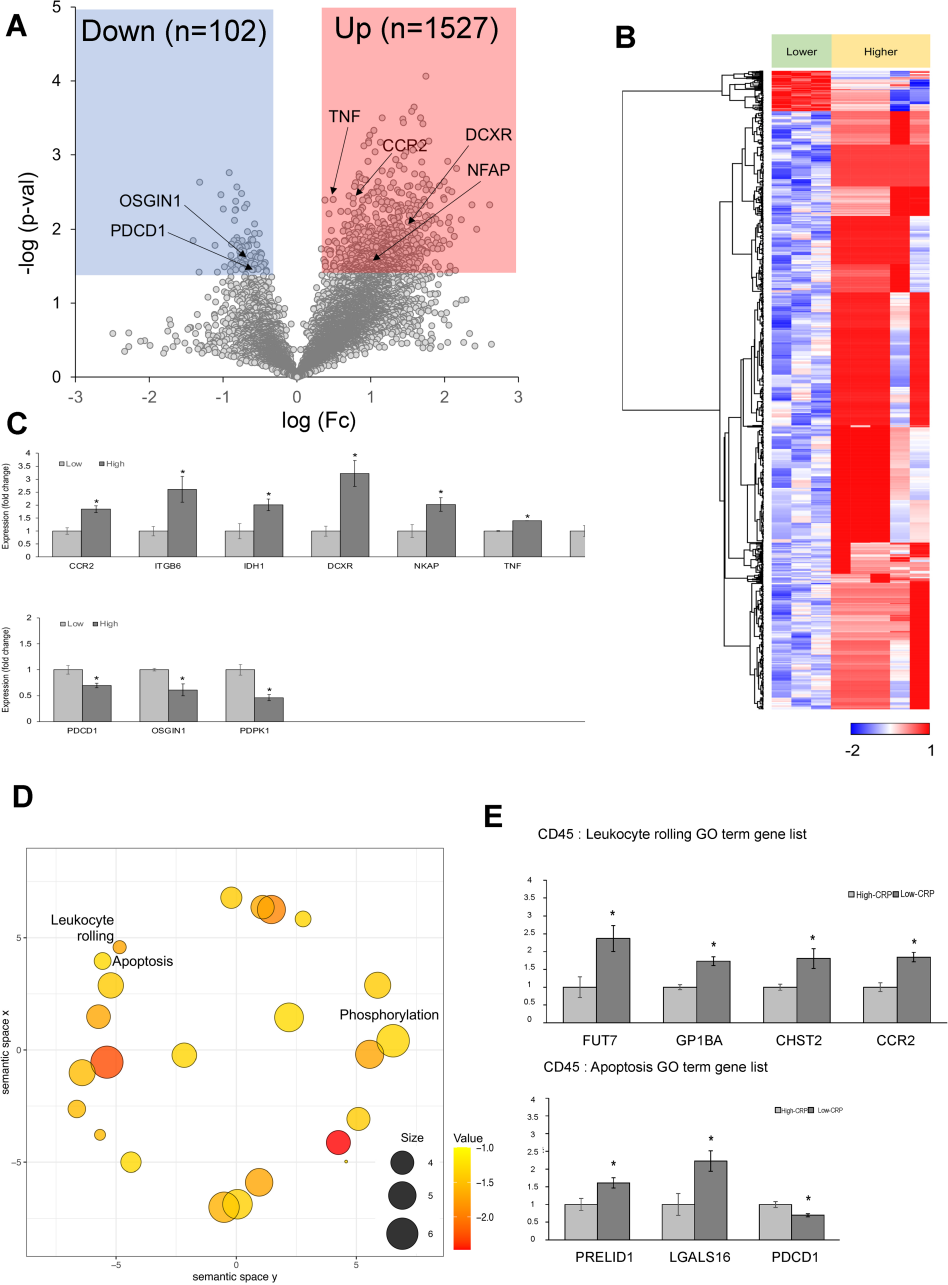
Differentially expressed genes (DEGs) in CD45-positive lymphocytes from spatial whole transcriptomic analysis [AAA–high CRP (n = 7) versus AAA–low CRP (n = 3)]. **(A)** Volcano plot displaying highly expressed DEGs in CD45-positive lymphocytes based on CRP deposition in AAA, using log_10_ fold changes. **(B)** Clustered heatmap of the selected ROIs showing whole transcriptomic expression fold changes (average gene expression, z-score) in relation to CRP levels in AAA. A total of 1,602 genes were differentially expressed according to the CRP deposition level. **(C)** Key DEGs between AAA–high-CRP and AAA–low-CRP groups from **(A)**. **(D)** Scatter plot from REVIGO illustrating cluster representatives after redundancy reduction in a two-dimensional space. Notable GO terms identified include apoptosis and leukocyte rolling. The bubble size indicates the frequency of the GO terms in the underlying GOA database, with color shading representing user-supplied p-values (legend in the lower right corner). **(E)** GO analysis results of genes associated with “leukocyte rolling,” and “apoptosis.”. DEG, differentially expressed gene; Down, downregulated; Up, upregulated; AAA–high CRP, abdominal aortic aneurysm with high CRP; AAA–low CRP, abdominal aortic aneurysm with low CRP. The asterisk symbol (*) indicates statistical significance at p < 0.05.

In CD45-positive lymphocytes, approximately 1,629 DEGs were observed between the high- CRP and low-CRP groups (**Figures 5A, B**). Notably, the high-CRP group showed increases in *NKAP, TNF, CCR2, CCR6, ITGB6, DCXR*, and *IDH1* (**Figure 5C**). NKAP (NF-κB–activating protein) triggers macrophage infiltration and inflammation in the adventitia and media to induce vascular remodeling and contribute to the aneurysmal formation (35). TNF contributes to AAA formation by playing a central role in aortic wall inflammation and matrix remodeling by increasing pro-inflammatory cytokines and matrix metalloproteinase (MMP) expression (36, 37). CCR2 (C-C chemokine receptor type 2) recruits and activates monocytes and macrophages, increases inflammation in AAA, and promotes extracellular matrix degradation (38). CCR6 (CC chemokine receptor 6), which is highly expressed in the aneurysmal wall, contributes to aneurysm development by promoting the migration of T cells (39) and natural IL-17–producing γδ T cells (40). ITGB6 (integrin β6) promotes extracellular matrix degradation, inflammation, and fibrosis (41). Furthermore, genes involved in cellular metabolic processes, such as DCXR (dicarbonyl and L-xylulose reductase (42)) and *IDH1* (43), were also upregulated in the high-CRP group.

In the CD45-positive lymphocytes of the high-CRP group, the following genes were significantly decreased: *PDCD1* (programmed cell death protein 1 or PD-1), which is a negative regulator of inflammation that helps prevent pathological immune responses (44); *OSGIN1* (oxidative stress–induced growth inhibitor 1), which protects endothelial cells from oxidative stress (45); and *PDPK1* (3-phosphoinositide-dependent protein kinase 1), which is necessary for the maintenance of CD4+ FOXP3+ regulatory T cells, immune cell homeostasis, and the inhibition of effector T-cell function (46). Thus, immune regulatory genes, negative regulators, and protectors were decreased in the high-CRP group.

#### 3.4.2 GO analysis

REVIGO analysis of DEGs in CD45-positive lymphocytes identified significant biological process terms, including leukocyte rolling and apoptosis (**Figure 5D**). In terms of genes associated with the GO term “leukocyte rolling,” FUT7, GP1BA, CHST2, and CCR2 were significantly upregulated in CD45-positive lymphocytes of the high-CRP group. FUT7 [fucosyltransferase VII (47, 48)], which is crucial for immune cell extravasation; GP1BA (glycoprotein Ib alpha chain), essential for leukocyte rolling and platelet adhesion to damaged endothelium, playing a key role in thrombosis (49); CHST2 (carbohydrate sulfotransferase 2), important for leukocyte rolling (50); and CCR2, which is involved in monocyte and macrophage recruitment during inflammation and related to leukocyte rolling (51), were significantly upregulated (**Figure 5E**).

In terms of genes associated with the GO term “apoptosis,” the high-CRP group showed decreases in PRELID1 (PRELI domain–containing protein 1), which induces mitochondrial ROS production and apoptosis (52) and LGALS16 (galectin-16), a strong inducer of apoptosis in CD3+ T lymphocytes (53) (**Figure 5E**).

### 3.5 αSMA-positive vascular smooth muscle cells

#### 3.5.1 DEGs according to the level of CRP deposition

**Figure 6:**
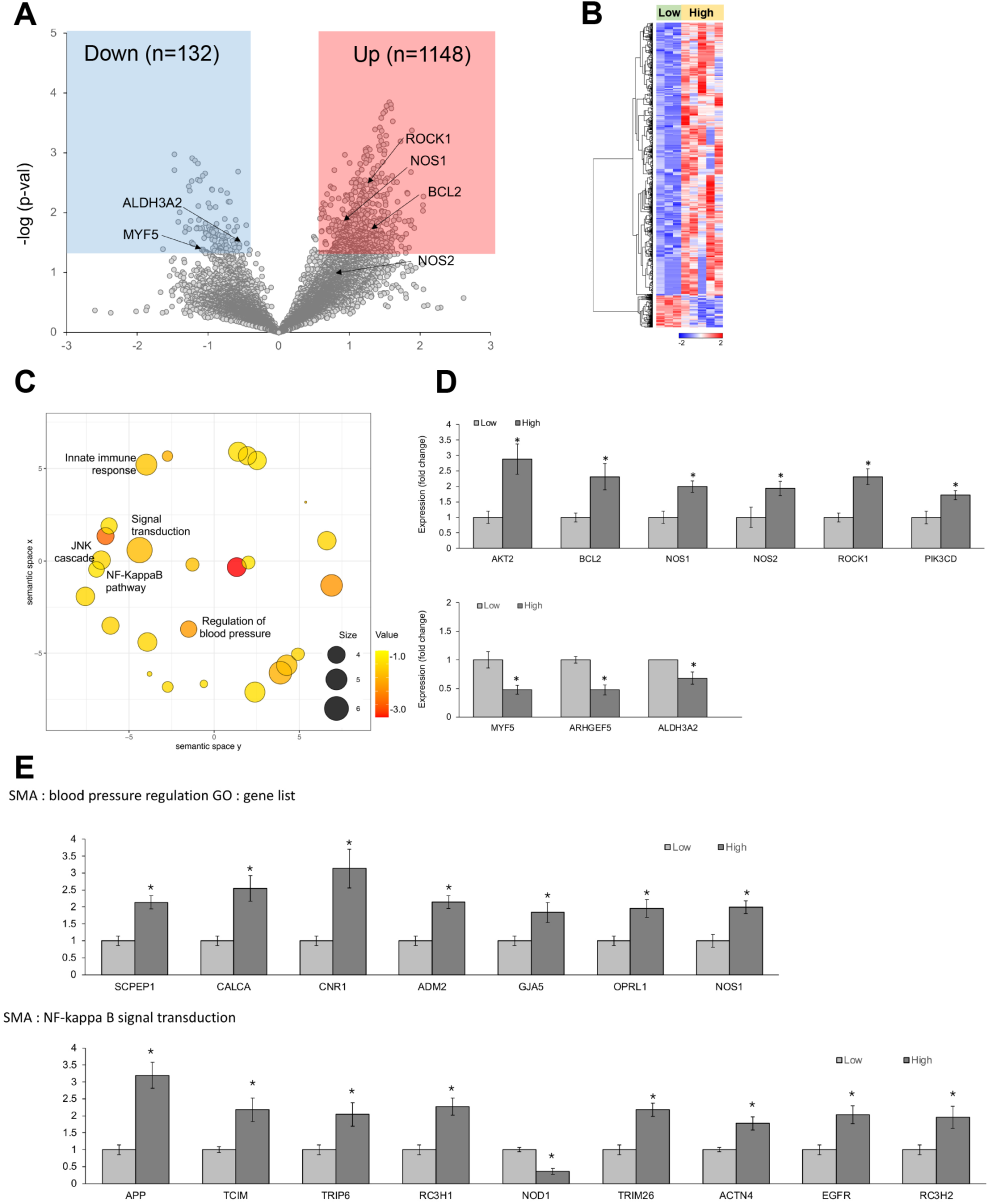
Differentially expressed genes (DEGs) in αSMA-positive vascular smooth muscle cells from spatial whole transcriptomic analysis [AAA–high CRP (n = 7) versus AAA–low CRP (n = 3)]. **(A)** Volcano plot displaying highly expressed DEGs in αSMA-positive vascular smooth muscle cells based on CRP deposition in AAA, using log_10_ fold changes. **(B)** Clustered heatmap of the selected ROIs showing whole transcriptomic expression fold changes (average gene expression, z-score) in relation to CRP levels in AAA. A total of 1,280 genes were differentially expressed according to the CRP deposition level. **(C)** Scatter plot from REVIGO illustrating cluster representatives after redundancy reduction in a two-dimensional space. Notable GO terms identified include NF-κB signal transduction, NJK cascade, and regulation of blood pressure. The bubble size indicates the frequency of the GO terms in the underlying GOA database, with color shading representing user-supplied p-values (legend in the lower left corner). **(D)** Key DEGs between the AAA–high-CRP and AAA–low-CRP groups from **(A)**. **(E)** GO analysis results of genes associated with “blood pressure regulation” and “NF-κB signal transduction.” DEG, differentially expressed gene; Down, downregulated; Up, upregulated; AAA–high CRP, abdominal aortic aneurysm with high CRP; AAA–low CRP, abdominal aortic aneurysm with low CRP. The asterisk symbol (*) indicates statistical significance at p < 0.05.

In αSMA-positive vascular smooth muscle cells (VSMCs), 1,280 genes exhibited differential expression depending on the CRP levels (**Figures 6A, B**). Specifically, *AKT2, BCL2, NOS1, NOS2, ROCK1, *and *PIK3CD* were upregulated in the high-CRP group (**Figure 6D**). AKT2 regulates vascular cell survival (54), endothelial NO synthase and vascular tone (55), and VSMC proliferation (56). BCL2 regulates apoptosis in VSMCs (57, 58). NOS1 and NOS2 generate NO and regulate vascular tone (59). ROCK1 (Rho-associated, coiled-coil containing protein kinase 1) regulates the contraction of VSMCs and the maintenance of vascular tone (60); accordingly, the dysregulation of ROCK1 can alter the phenotype of VSMCs, promote their proliferation, contribute to the formation of neointima, and facilitate vascular remodeling (61). PIK3CD (phosphoinositide-3-kinase catalytic subunit delta) is involved in the proliferation, survival, and migration of VSMCs (62).

Conversely, the AAA–high-CRP group showed a decrease in the level of *ALDH3A2* (aldehyde dehydrogenase 3 family member A2), which is involved in detoxifying lipid peroxidation-derived aldehydes and plays a role in lipid metabolism by oxidizing fatty aldehydes to fatty acids (63) (**Figure 6D**).

#### 3.5.2 GO analysis

GO analysis of the upregulated genes in αSMA from the high-CRP group revealed significant alterations in the JNK cascade, NF-κB signaling pathway, and the regulation of the blood pressure pathway (**Figure 6C**). Notably, genes associated with the NF-κB signaling pathway, including *APP, TCIM, TRIP6, TRIM26, ACTN4, EGFR,* and *RC3H2,* were significantly increased, whereas *NOD1* was significantly decreased (**Figure 6E**).

Within the blood pressure regulation category, genes such as *SCPEP1, CALCA, CNR1, ADM2, GJA5, OPRL1,* and *NOS1 *were significantly upregulated in the AAA–high-CRP group (**Figure 6E**).

*SCPEP1* (serine carboxypeptidase 1) is involved in the regulation of vascular tone and hemodynamics. By inactivating endothelin-1 through cleavage of its C-terminal Trp residue, SCPEP1 promotes vasoconstriction, and its upregulation increases vascular sensitivity to ET-1, leading to elevated blood pressure (64). CALCA (calcitonin gene–related peptide alpha) is a potent microvascular vasodilator (65), is considered a therapeutic target for subarachnoid hemorrhage-triggered cerebral vasospasm and is known to play a protective role in vascular diseases such as hypertension (66); however, its direct role in aortic aneurysms remains to be elucidated. CNR1 (cannabinoid receptor 1, CB1) plays a role in vasodilation, reducing vascular resistance, and lowering blood pressure. CB1 antagonists exert beneficial effects in atherosclerosis and restenosis by decreasing vascular smooth muscle proliferation and migration (67). ADM2 (adrenomedullin 2) exerts a vasomotor effect by inducing peripheral vasodilation through binding to its receptors on endothelial cells and VSMCs (68, 69). GJA5 (gap junction protein alpha 5, connexin 40) is crucial for coordinating endothelial and smooth muscle cell functions, and it regulates vasomotor tone by transmitting electrical signals from the endothelium to the underlying smooth muscle and moderates arterial blood pressure through the renin-angiotensin system in afferent arterioles (70). The downregulation of connexin 40 has been associated with the development of aortic aneurysms (71). Furthermore, NOS1 (neuronal-derived NOS) exerts a potent vasodilatory effect (72).

### 3.6 Validation using pSTAT3 immunohistochemistry

**Figure 7:**
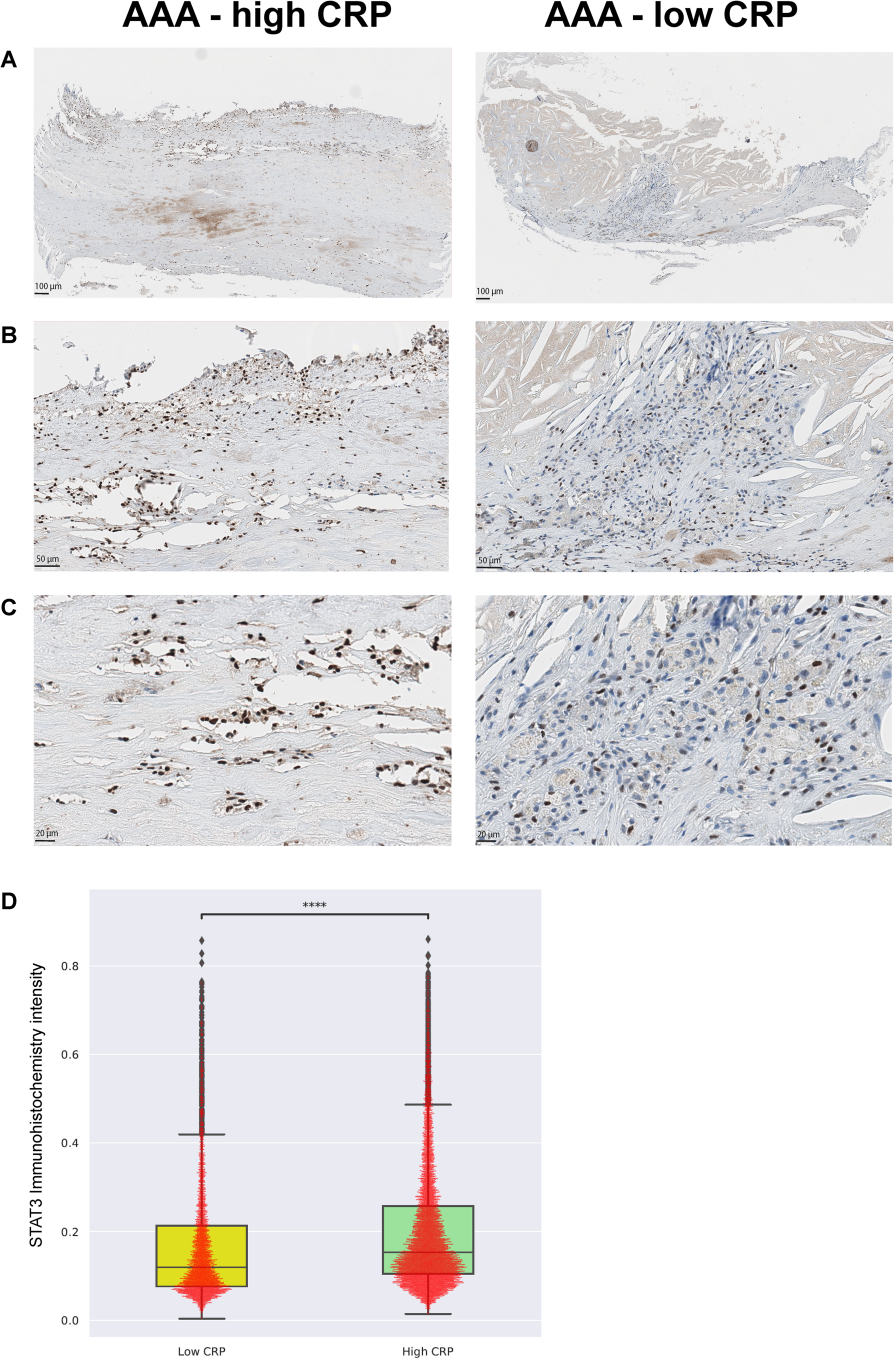
Validation with phospho-STAT3 immunohistochemistry [AAA-high CRP (n = 7) versus AAA-low CRP (n = 3)]. To validate the transcriptome enrichment of STAT3 in AAA–high CRP compared to that in AAA–low CRP, immunohistochemical staining for phospho-STAT3 was performed. **(A)** Low magnification, **(B)** medium magnification, and **(C)** high magnification. **(D)** Intensity of nuclear pSTAT3-positive cells in the vascular tissue of the AAA-high-CRP group compared to that of the AAA–low-CRP group (Mann–Whitney–Wilcoxon test two-sided with Bonferroni correction, p < 0.0001). The symbols used to indicate levels of statistical significance are as follows: *p < 0.05, **p < 0.01, ***p < 0.001, and ****p < 0.0001.

Immunohistochemistry targeting phosphorylated STAT3 (pSTAT3) was performed to verify its expression in CD68-positive macrophages, which are central to inflammation, apoptosis, chemotaxis, type 1 T helper cell response, and defense response signaling. Consistent with the spatial transcriptomic results, the high-CRP group showed a significant increase in pSTAT3 expression (p < 0.001) (**Figure 7**).

## 4 Discussion

The present study investigated the lineage-specific transcriptome of AAA tissue in relation to the degree of CRP deposition by employing GeoMx for spatially resolved whole RNA transcriptome analysis. CRP deposition was associated with numerous transcriptomic alterations in macrophages, lymphocytes, and VSMCs, which may be linked to the pathogenesis of AAA. In CD68-positive macrophages, we identified significant dysregulations in pathways related to the inflammatory response, regulation of the MAPK pathway, and maintenance of blood vessel diameter. In CD45-positive lymphocytes, pathways related to leukocyte rolling and apoptosis were prominently highlighted. Additionally, in αSMA-positive smooth muscle cells, significantly upregulated pathways included NF-κB signaling transduction, the JNK cascade, and regulation of blood pressure.

Recent research by Fu et al. demonstrated that CRP-deficient mice exhibited suppressed aneurysmal elastin destruction in the aorta when AAA was induced with elastase (73). In our previous research, we reported extensive proteomic changes in tissues with strong CRP deposition between atheroma and eroded aortic aneurysmal tissue in AAA; moreover, our CODEX multiplexed tissue imaging using 31 antibodies for deep phenotyping revealed pro-inflammatory immune cell deposition and M1 macrophage polarization in CRP-deposited aortic walls (13). Combining our findings with those of Fu et al., we suggest that CRP deposition is closely related to the pathogenesis of AAA in that its accumulation in the aortic wall may actively drive the pathogenesis and progression by modulating the cellular landscape.

In the current study, by employing spatial transcriptomics, we were able to analyze gene expression separately for each cellular lineage and in relation to the degree of CRP deposition. This approach allowed us to identify gene dysregulation that had not been detected in previous studies using bulk sequencing on aneurysm-specific samples. A previous study analyzed the full-length transcriptome of AAA using nanopore-based direct RNA sequencing (74); however, this study lacked spatial information. Our study is the first to conduct a whole transcriptomic analysis while preserving spatial information.

In this study, patients with AAA with a high degree of CRP deposition showed significant inflammatory responses, apoptosis, chemotaxis, type 1 T helper cell immune response, and defense response signaling in macrophages and leukocytes. Recently, there has been increasing interest and research into the active pro-inflammatory role of CRP. In the serum, CRP exists in a pentameric form, where it plays a protective role by promoting opsonization and assisting phagocytes in clearing bacteria or damaged cells, thus preventing further damage. However, when CRP encounters damaged cell membranes or macrovesicles, it undergoes a structural change from the pentameric form to the monomeric form (mCRP). This transformation enhances pro-inflammatory processes, leading to the activation of endothelial cells, monocytes, platelets, and neutrophils. It also promotes the adhesion of inflammatory cells to endothelial cells, strengthens neutrophil-platelet and platelet-monocyte aggregation, and increases the production of pro-inflammatory cytokines, thereby contributing to tissue damage (75, 76). Accordingly, we observed enhanced pro-inflammatory signaling, innate immune response, and leukocyte rolling signaling in the CRP-deposited areas of AAA tissue. These findings suggest that CRP deposition in AAA may enhance pro-inflammatory signals, thereby exacerbating tissue damage in AAA.

Recent studies have identified inflammation and immune response as key factors in the pathogenesis of AAA. Established risk factors, such as hypercholesterolemia, hypertension, and cigarette smoking, trigger vascular endothelial injury, which promotes leukocyte infiltration and initiates inflammatory cascades. Monocyte infiltration into the vessel wall leads to lipid accumulation and foam cell formation, whereas T lymphocytes accumulate in the intimal layer, releasing interferon-gamma and TNF. These cytokines further activate macrophages, as well as vascular endothelial and smooth muscle cells, perpetuating the inflammatory state. This chronic inflammation exacerbates endothelial injury and promotes extracellular matrix remodeling, driving the progression of AAA (5, 77). Our findings underscore the significant role of CRP deposition within the aortic wall as a potent pro-inflammatory stimulus, suggesting that it may amplify these pathological processes by fostering a chronic inflammatory environment critical to the development and progression of AAA.

Five genes—*STAT3, IL-17A, IFI16, ITGB2, *and *SPI1*—were notably upregulated in the high-CRP group. Especially, we found that pro-inflammatory and pro-apoptotic signals were centrally connected via STAT3, suggesting that STAT3 plays a central role in mediating aortic wall pathophysiologic changes associated with CRP deposition. STAT3 is essential for inflammation and is well-known as a key mediator in the pathogenesis and progression of AAA (78, 79). IL1-7A, which is mediated by the activation of STAT3, induces aneurysm development by the production of pro-inflammatory cytokines in macrophages through the MAP kinase, NF-κB, and AP-1 pathways (80), thereby influencing macrophages and activating atherogenesis (81). Inhibition of STAT3 has been shown to prevent the progression of AAA (82, 83). Additionally, IL-6, a major inducer of CRP expression, also activates STAT3 signaling (84). There is evidence that STAT3 participates in the transcriptional activation of CRP in response to IL-6 (85). Furthermore, studies have identified multiple STAT3-binding sites on the promoter region of the *CRP* gene (positions 72, 108, 134, and 164) (86). Therefore, there might be a close crosstalk between CRP deposition and STAT3. This synergistic effect between CRP and STAT3 likely enhances pro-inflammatory and pro-apoptotic signals, exacerbating AAA pathology.

IFI16 (interferon gamma–inducible protein 16) is involved in pro-inflammatory responses, DNA sensing, and interferon signaling. ITGB2 (integrin subunit beta 2) plays a crucial role in neutrophil extravasation and macrophage complement receptor function. SP1 [Spi-1 proto-oncogene, transcription factor PU.1 (87)] plays a role in cell fate decisions and pro-fibrotic activities. The increase in pro-fibrotic SP1 in macrophages of the high-CRP group is likely associated with increased fibrosis related to atherosclerosis.

We found that AAA tissues with a high degree of CRP deposition had downregulations in the expression of CXCL8 and CNR2, which are associated with M2 macrophage polarization (22). Additionally, PGFER1, which fine-tunes M1-M2 macrophage polarization, was also downregulated, suggesting a shift toward M1 polarization in macrophages in the presence of CRP deposition. Other studies have also reported that CRP induces M1 macrophage polarization and inhibits the transformation to the M2 phenotype, thereby enhancing the inflammatory response (88, 89). Our previous research using CODEX multiplexed imaging on CRP-deposited AAA tissues also demonstrated that high–CRP deposition levels were associated with M1 macrophage polarization, while low–CRP deposition levels were associated with M2 polarization. Moreover, M1 polarization of macrophages in the aortic wall has been shown to exacerbate aortic aneurysm formation (90). Collectively, these findings suggest that CRP deposition in the aortic wall is associated with a pro-inflammatory milieu through M1 macrophage polarization, which may contribute to the pathogenesis of AAA.

NF-κB, MAPK, and JAK-STAT pathways closely interact with each other and are involved in the development of aortic aneurysms (35, 91). NF-κB plays a prominent role in atherosclerosis and is also a key element in the pathogenesis of aortic aneurysms (92). Specifically, NF-κB enhances endothelial cell dysfunction and inflammation (35), increases pro-inflammatory cytokines, and promotes the infiltration of lymphocytes and macrophages from the vascular lumen into the subendothelial layer of the arterial intima (35, 91, 93), thereby contributing to the development of aortic aneurysms.

The MAPK pathway, particularly the ERK pathway, plays a crucial role in the development of AAA by regulating the activation of MMPs (94). The JNK cascade, a component of the MAPK pathway, activates MMP9, which is essential for AAA formation (95–97). Inhibiting the JNK pathway has been shown to prevent aortic aneurysms (95), highlighting its potential as a therapeutic target for AAA. In this study, we found that CRP deposition significantly activated the MAPK pathway in macrophages and the NF-κB and JNK pathways in VSMCs. These pathways were markedly more active in the high-CRP group compared to those in the low-CRP group.

Interestingly, we found that a high degree of CRP deposition in AAA was associated with significant downregulation of blood vessel diameter maintenance–related genes in macrophages. Furthermore, genes associated with vasodilation and protective roles were predominantly upregulated in the high-CRP group. These results suggest that CRP-induced transcriptomic changes are closely linked to the development and exacerbation of AAA. Whether this upregulation of vasodilatory genes acts protectively against aneurysm formation or contributes to a deleterious effect by weakening the vasoconstrictive strength of the aorta and thus promoting the progression of aortic aneurysms requires further investigation.

### 4.1 Source of CRP deposited in AAA

Some reports suggested that certain types of macrophages express CRP (98), and Kaplan et al. (99) reported that, in aortic aneurysms, macrophages express CRP, which leads to macrophage polarization toward the M1 phenotype through the NF-κB signaling pathway. In this study, however, CRP transcriptomes were not detected in macrophages, lymphocytes, or VSMC components. Therefore, it is suggested that the CRP deposited in the vascular wall is not secreted by macrophages but likely originates from serum CRP, which deposits onto the atherosclerotic wall. Our previous studies (12) demonstrated a correlation between serum CRP levels and the immunopositivity of CRP deposited in atherosclerotic plaques. This supports the idea that, in AAA, CRP is deposited from circulating blood rather than produced within the vascular wall.

### 4.2 Relationship between atherosclerosis, aortic aneurysm, and CRP

Atherosclerosis is closely linked to the pathophysiology of aneurysms. A detailed pathological study conducted by Xu et al. revealed that, in aortic walls with significant plaque accumulation, the internal elastic lamina adjacent to the plaque is lost. As this condition progresses, the media thins and results in the formation of an aneurysm (100). Furthermore, AAA and atherosclerosis share clinical risk factors and molecular mechanisms, including male sex, tobacco use, family history, hyperlipidemia, and advanced age (101). Both conditions are characterized by chronic inflammation and accompanying immune responses.

However, there are distinct clinical features that differentiate aortic aneurysms from atherosclerosis. For instance, whereas atherosclerotic disease is common in patients with diabetes mellitus, aortic aneurysms are conversely less prevalent in this population, suggesting a protective effect (102, 103). Therefore, while atherosclerosis and aortic aneurysms are closely related and share similar etiologies and progression, the direct causal relationship between the two conditions has not been well-defined. Nonetheless, the significant overlap between these diseases indicates that there is still much to be understood about their close association in certain subsets of patients.

Our current results suggest that CRP deposition may play a role in the link between atherosclerosis and aneurysms. In our previous study (12), using IHC antibody specific to the mCRP form, we observed strong mCRP immunodeposition at the interface of the eroded aortic wall in both atherosclerosis and aortic aneurysms. CRP immunopositivity was predominantly observed in atherosclerotic regions. Proteomic analysis of this area revealed significant activation of pro-inflammatory signaling, complement signaling, and thrombosis signaling pathways. Therefore, our results suggest that the deposition of CRP in various components of AAA led to widespread transcriptome dysregulation, exacerbating the condition.

### 4.3 Comparison with published single-cell RNA sequencing

Recent studies have provided single-cell RNA sequencing (scRNA-seq) data on AAA. However, most studies have primarily focused on the pathogenesis of AAA, particularly utilizing induced animal models. In contrast, our study investigated the transcriptomic differences within AAA lesions based on the extent of CRP deposition. Consequently, direct comparisons with studies that analyze AAA in relation to normal aorta are limited. Nonetheless, a common finding across the majority of studies is the central role of inflammation in the progression of AAA, with particular emphasis on the crucial contribution of macrophages. Le et al. (104) reported in an angiotensin II–infused ApoE−/− mouse model of AAA that gene set variation analysis (GSVA) of scRNA-seq data revealed activation of the JAK-STAT pathway in monocytes and macrophages, promoting the secretion of type I interferon. These monocytes and macrophages were identified as interferon-inducible (IFNIC), contributing to the development of AAA. Similarly, in our study, CD68+ macrophages in CRP-enriched AAA lesions exhibited elevated JAK-STAT pathway activity, suggesting a comparable role in AAA progression. Both studies highlight the involvement of the JAK-STAT pathway in macrophages during AAA progression. Additionally, Le et al. also observed activation of the NF-κB signaling pathway, as we did, further reinforcing the inflammatory nature of aneurysm development. Given that CRP deposition in our study correlates with enhanced expression of these pathways, we propose that CRP contributes to the progression of inflammation-induced aneurysms.

Zhang et al. (105) found that, while normal controls clustered homogeneously, AAA samples formed multiple distinct clusters, suggesting the presence of subgroups within AAA. Our findings similarly indicate that CRP depositions may stratify AAA into distinct subgroups, further supporting the role of CRP in the pathogenesis of AAA. Additionally, Zhang’s study identified CXCL8 as a key gene associated with AAA, which aligns with our findings, where leukocyte rolling and related pathways were significantly enriched. Yuan et al. (106) analyzed CD45+ cell populations in an elastase-induced murine model of AAA and observed increased expression of inducible nitric oxide synthase (iNOS) in CD68+ macrophages and CCR6+ CD4+ T cells in AAA compared to that in normal tissue. In our study, these gene expressions were significantly upregulated in CRP-enriched AAAs, further reinforcing the relationship between CRP deposition and enhanced inflammatory responses in AAA. Yuan et al. also reported the enrichment of interferon-induced proteins, including Ifit1, in response to interferon signaling (106), which aligns with our observations regarding the pivotal role of macrophages in CRP-rich AAAs. Moreover, their pseudo-time analysis indicated a shift in monocyte/macrophage phenotypes driven by unidentified stimuli, and we propose that CRP could be a potential factor contributing to these transcriptomic alterations in macrophages. Xiong et al. (107) reported the upregulation of the PI3K-AKT signaling pathway in macrophages within mouse AAA samples, which is consistent with our findings of elevated PI3K-AKT signaling in VSMCs. In our study, the TNF signaling pathway was upregulated in CD45+ lymphocytes within CRP-rich AAAs, whereas Xiong’s study observed its activation in fibroblasts.

### 4.4 Limitations

Firstly, due to IRB issues, we utilized samples that had been archived for over 10 years. However, all samples passed QC and yielded biologically relevant results consistent with previous IHC (12) and multiplexed imaging findings (13). This confirms the feasibility of performing spatial transcriptomic analysis on long-archived FFPE paraffin blocks. Secondly, during STAT3 validation, we were unable to specifically quantify its expression in macrophages. Future studies should incorporate multiplexed tissue imaging for a more detailed analysis in this context.

A further limitation of this study is the use of thoracic aorta specimens, obtained during heart transplantation, as controls. Given the difficulty of obtaining normal abdominal aorta tissue from living patients, thoracic aorta samples were used despite their distinct embryological origins—where the thoracic aorta derives from neural crest cells (108) and the abdominal aorta originates from mesodermal tissue (109). This difference may introduce some bias. To address this issue, our primary analysis concentrated on the differential gene expression between the CRP-low and CRP-high groups, thereby minimizing reliance on comparisons with the normal control group.

Another limitation of this study is the difficulty in determining whether the observed transcriptomic changes are directly attributable to CRP deposition or are secondary to inflammation, as CRP deposition often follows IL-6–driven inflammatory processes. In studies of human diseases, it is inherently difficult to separate the direct effects of CRP from the broader inflammatory response, as these processes typically occur concurrently. However, in our previous study utilizing a rat model of ischemia-reperfusion myocardial injury, we injected CRP in the absence of an inflammatory environment (110, 111). We observed that CRP accumulated in ischemic myocardial cells, exacerbating damage, increasing infarct size (110), and inducing widespread changes in microRNA expression (111). This suggests that CRP deposition itself, rather than inflammation-induced processes, can intensify damage in already compromised cells (76). To further elucidate this distinction, future *in vitro* studies, including CRP loss-of-function experiments, are necessary to isolate the specific effects of CRP.

## 5 Conclusions

**Figure 8:**
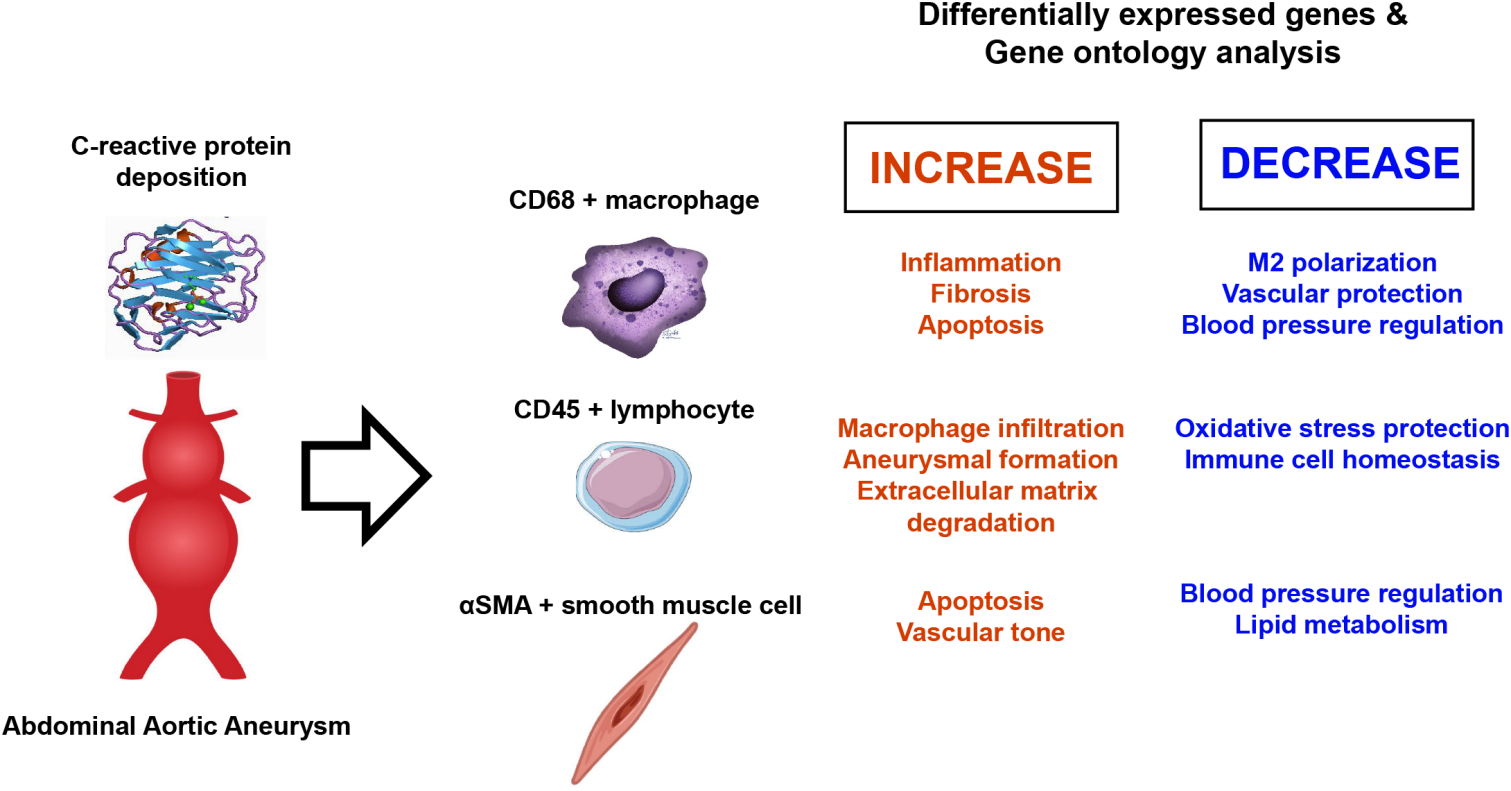
Gene expression changes in abdominal aortic aneurysm.

CRP deposition in aortic atheroma was associated with significant changes in the transcriptomic profile of macrophages, promoting pro-inflammatory and apoptotic alterations predominantly mediated by STAT3. Furthermore, CRP deposition affected genes involved in the MAPK and JNK signaling pathways, vascular diameter regulation, and smooth muscle blood pressure signaling pathways (**Figure 8**). These findings suggest that CRP is not merely a prognostic marker for AAA but also plays an active role in its pathogenesis at the transcriptomic level. Thus, targeting CRP-associated pathways could present novel therapeutic strategies for managing AAA.

## Data Availability

The datasets presented in this study can be found in online repositories. The names of the repository/repositories and accession number(s) can be found below: PRJNA1146589 (SRA).

## References

[1] Aggarwal S Qamar A Sharma V Sharma A. Abdominal aortic aneurysm: A comprehensive review. Exp Clin Cardiol. (2010) 16:11–5. PMC3076160 21523201

[2] Golledge J. Abdominal aortic aneurysm: update on pathogenesis and medical treatments. Nat Rev Cardiol. (2019) 16:225–42. doi: 10.1038/s41569-018-0114-9 30443031

[3] Sampson UKA Norman PE Fowkes FGR Aboyans V Song Y Harrell FE. Global and regional burden of aortic dissection and aneurysms mortality trends in 21 world regions, 1990 to 2010. Global Hear. (2014) 9:171–180.e10. doi: 10.1016/j.gheart.2013.12.010 25432126

[4] Golledge J Moxon JV Singh TP Bown MJ Mani K Wanhainen A. Lack of an effective drug therapy for abdominal aortic aneurysm. J Intern Med. (2020) 288:6–22. doi: 10.1111/joim.12958 31278799

[5] Márquez-Sánchez AC Koltsova EK. Immune and inflammatory mechanisms of abdominal aortic aneurysm. Front Immunol. (2022) 13:989933. doi: 10.3389/fimmu.2022.989933 36275758 PMC9583679

[6] Kuivaniemi H Ryer EJ Elmore JR Tromp G. Understanding the pathogenesis of abdominal aortic aneurysms. Expert Rev Cardiovasc Ther. (2015) 13:975–87. doi: 10.1586/14779072.2015.1074861 PMC4829576 26308600

[7] Haro JD Bleda S Acin F. C-reactive protein predicts aortic aneurysmal disease progression after endovascular repair. Int J Cardiol. (2016) 202:701–6. doi: 10.1016/j.ijcard.2015.09.122 26454539

[8] Schillinger M Domanovits H Bayegan K Hölzenbein T Grabenwöger M Thoenissen J. C-reactive protein and mortality in patients with acute aortic disease. Intensiv Care Med. (2002) 28:740–5. doi: 10.1007/s00134-002-1299-1 12107680

[9] Haro JD Acin F Bleda S Varela C Medina FJ Esparza L. Prediction of asymptomatic abdominal aortic aneurysm expansion by means of rate of variation of C-reactive protein plasma levels. J Vasc Surg. (2012) 56:45–52. doi: 10.1016/j.jvs.2012.01.003 22551908

[10] Braig D Nero TL Koch H-G Kaiser B Wang X Thiele JR. Transitional changes in the CRP structure lead to the exposure of proinflammatory binding sites. Nat Commun. (2017) 8:14188. doi: 10.1038/ncomms14188 28112148 PMC5264208

[11] Dix C Zeller J Stevens H Eisenhardt SU Shing KSCT Nero TL. C-reactive protein, immunothrombosis and venous thromboembolism. Front Immunol. (2022) 13:1002652. doi: 10.3389/fimmu.2022.1002652 36177015 PMC9513482

[12] Kim EN Yu J Lim JS Jeong H Kim CJ Choi J-S. CRP immunodeposition and proteomic analysis in abdominal aortic aneurysm. PloS One. (2020) 16:e0245361. doi: 10.1371/journal.pone.0245361 PMC8384196 34428207

[13] Kim EN Seok HY Koh J Yang W Lee GH Choi WH. Unraveling the complexity of abdominal aortic aneurysm: multiplexed imaging insights into C-reactive protein-related variations. bioRxiv. (2024). doi: 10.1101/2024.02.22.581315. 2024.02.22.581315.

[14] Lee S-Y Kim MY Han JH Park SS Yun Y Jee S-C. Ramifications of POU4F3 variants associated with autosomal dominant hearing loss in various molecular aspects. Sci Rep. (2023) 13:12584. doi: 10.1038/s41598-023-38272-w 37537203 PMC10400627

[15] Love MI Huber W Anders S. Moderated estimation of fold change and dispersion for RNA-seq data with DESeq2. Genome Biol. (2014) 15:550. doi: 10.1186/s13059-014-0550-8 25516281 PMC4302049

[16] Huang DW Sherman BT Lempicki RA. Systematic and integrative analysis of large gene lists using DAVID bioinformatics resources. Nat Protoc. (2009) 4:44–57. doi: 10.1038/nprot.2008.211 19131956

[17] Supek F Bošnjak M Škunca N Šmuc T. REVIGO summarizes and visualizes long lists of gene ontology terms. PloS One. (2011) 6:e21800. doi: 10.1371/journal.pone.0021800 21789182 PMC3138752

[18] Szklarczyk D Gable AL Lyon D Junge A Wyder S Huerta-Cepas J. STRING v11: protein–protein association networks with increased coverage, supporting functional discovery in genome-wide experimental datasets. Nucleic Acids Res. (2019) 47:D607–13. doi: 10.1093/nar/gky1131 PMC6323986 30476243

[19] Bankhead P Loughrey MB Fernández JA Dombrowski Y McArt DG Dunne PD. QuPath: Open source software for digital pathology image analysis. Sci Rep. (2017) 7:16878. doi: 10.1038/s41598-017-17204-5 29203879 PMC5715110

[20] An J Naruse TK Hinohara K Soejima Y Sawabe M Nakagawa Y. MRTF-A regulates proliferation and survival properties of pro-atherogenic macrophages. J Mol Cell Cardiol. (2019) 133:26–35. doi: 10.1016/j.yjmcc.2019.05.015 31128166

[21] Hinkel R Trenkwalder T Petersen B Husada W Gesenhues F Lee S. MRTF-A controls vessel growth and maturation by increasing the expression of CCN1 and CCN2. Nat Commun. (2014) 5:3970. doi: 10.1038/ncomms4970 24910328

[22] Shao Y Lan Y Chai X Gao S Zheng J Huang R. CXCL8 induces M2 macrophage polarization and inhibits CD8+ T cell infiltration to generate an immunosuppressive microenvironment in colorectal cancer. FASEB J. (2023) 37:e23173. doi: 10.1096/fj.202201982rrr 37665572

[23] Tortora C Paola AD Argenziano M Creoli M Marrapodi MM Cenni S. Effects of CB2 receptor modulation on macrophage polarization in pediatric celiac disease. Biomedicines. (2022) 10:874. doi: 10.3390/biomedicines10040874 35453624 PMC9029516

[24] Du Y Ren P Wang Q Jiang S-K Zhang M Li J-Y. Cannabinoid 2 receptor attenuates inflammation during skin wound healing by inhibiting M1 macrophages rather than activating M2 macrophages. J Inflammation. (2018) 15:25. doi: 10.1186/s12950-018-0201-z PMC6278147 30534003

[25] Mena HA Spite M. Proresolving receptor tames inflammation in atherosclerosis. J Clin Investig. (2021) 131:e155240. doi: 10.1172/jci155240 34907914 PMC8670831

[26] Arnardottir H Thul S Pawelzik S-C Karadimou G Artiach G Gallina AL. The resolvin D1 receptor GPR32 transduces inflammation-resolution and atheroprotection. J Clin Investig. (2021) 131 (24):e142883. doi: 10.1172/jci142883 34699386 PMC8670838

[27] Ngo VL Abo H Kuczma M Szurek E Moore N Medina-Contreras O. IL-36R signaling integrates innate and adaptive immune-mediated protection against enteropathogenic bacteria. Proc Natl Acad Sci. (2020) 117:27540–8. doi: 10.1073/pnas.2004484117 PMC7959549 33087566

[28] Holditch SJ Schreiber CA Burnett JC Ikeda Y. Arterial remodeling in B-type natriuretic peptide knock-out females. Sci Rep. (2016) 6:25623. doi: 10.1038/srep25623 27162120 PMC4861904

[29] Liljeqvist ML Hultgren R Bergman O Villard C Kronqvist M Eriksson P. Tunica-specific transcriptome of abdominal aortic aneurysm and the effect of intraluminal thrombus, smoking, and diameter growth rate. Arter Thromb Vasc Biol. (2020) 40:2700–13. doi: 10.1161/atvbaha.120.314264 32907367

[30] Moyes AJ Khambata RS Villar I Bubb KJ Baliga RS Lumsden NG. Endothelial C-type natriuretic peptide maintains vascular homeostasis. J Clin Investig. (2014) 124:4039–51. doi: 10.1172/jci74281 PMC4151218 25105365

[31] Cao D Khan Z Li X Saito S Bernstein EA Victor AR. Macrophage angiotensin-converting enzyme reduces atherosclerosis by increasing peroxisome proliferator-activated receptor α and fundamentally changing lipid metabolism. Cardiovasc Res. (2023) 119:1825–41. doi: 10.1093/cvr/cvad082 PMC10681664 37225143

[32] Russell FD. Urotensin II in cardiovascular regulation. Vasc Heal Risk Manag. (2008) 4:775–85. doi: 10.2147/vhrm.s1983 PMC2597773 19065995

[33] Osadchii OE. Emerging role of neurotensin in regulation of the cardiovascular system. Eur J Pharmacol. (2015) 762:184–92. doi: 10.1016/j.ejphar.2015.05.025 26004530

[34] Maghsoudi S Shuaib R Bastelaere BV Dakshinamurti S. Adenylyl cyclase isoforms 5 and 6 in the cardiovascular system: complex regulation and divergent roles. Front Pharmacol. (2024) 15:1370506. doi: 10.3389/fphar.2024.1370506 38633617 PMC11021717

[35] Saito T Hasegawa Y Ishigaki Y Yamada T Gao J Imai J. Importance of endothelial NF-κB signalling in vascular remodelling and aortic aneurysm formation. Cardiovasc Res. (2013) 97:106–14. doi: 10.1093/cvr/cvs298 23015640

[36] Griepke S Grupe E Lindholt JS Fuglsang EH Steffensen LB Beck HC. Selective inhibition of soluble tumor necrosis factor signaling reduces abdominal aortic aneurysm progression. Front Cardiovasc Med. (2022) 9:942342. doi: 10.3389/fcvm.2022.942342 36186984 PMC9523116

[37] Xiong W MacTaggart J Knispel R Worth J Persidsky Y Baxter BT. Blocking TNF-α Attenuates aneurysm formation in a murine model. J Immunol. (2009) 183:2741–6. doi: 10.4049/jimmunol.0803164 PMC4028114 19620291

[38] Zhang J Chen H Liu L Sun J Shi MA Sukhova GK. Chemokine (C-C motif) receptor 2 mediates mast cell migration to abdominal aortic aneurysm lesions in mice. Cardiovasc Res. (2012) 96:543–51. doi: 10.1093/cvr/cvs262 PMC3500042 22871590

[39] Zhang H-F Zhao M-G Liang G-B Yu C-Y He W Li Z-Q. Dysregulation of CD4+ T cell subsets in intracranial aneurysm. DNA Cell Biol. (2016) 35:96–103. doi: 10.1089/dna.2015.3105 26667180

[40] Arunachalam P Ludewig P Melich P Arumugam TV Gerloff C Prinz I. CCR6 (CC chemokine receptor 6) is essential for the migration of detrimental natural interleukin-17–producing &ggr;&dgr; T cells in stroke. Stroke. (2017) 48:1957–65. doi: 10.1161/strokeaha.117.016753 28611085

[41] Zhang Z Wang Z Liu T Tang J Liu Y Gou T. Exploring the role of ITGB6: fibrosis, cancer, and other diseases. Apoptosis. (2024) 29:570–85. doi: 10.1007/s10495-023-01921-6 38127283

[42] Ebert B Kisiela M Maser E. Human DCXR – another ‘moonlighting protein’ involved in sugar metabolism, carbonyl detoxification, cell adhesion and male fertility? Biol Rev. (2015) 90:254–78. doi: 10.1111/brv.12108 24720935

[43] Ni Y Shen P Wang X Liu H Luo H Han X. The roles of IDH1 in tumor metabolism and immunity. Futur Oncol. (2022) 18:3941–53. doi: 10.2217/fon-2022-0583 36621781

[44] Qin W Hu L Zhang X Jiang S Li J Zhang Z. The diverse function of PD-1/PD-L pathway beyond cancer. Front Immunol. (2019) 10:2298. doi: 10.3389/fimmu.2019.02298 31636634 PMC6787287

[45] Khoi C-S Xiao C-Q Hung K-Y Lin T-Y Chiang C-K. Oxidative stress-induced growth inhibitor (OSGIN1), a target of X-box-binding protein 1, protects palmitic acid-induced vascular lipotoxicity through maintaining autophagy. Biomedicines. (2022) 10:992. doi: 10.3390/biomedicines10050992 35625730 PMC9138516

[46] Oh H Zhao J Grinberg-Bleyer Y Postler TS Wang P Park S-G. PDK1 is required for maintenance of CD4+ Foxp3+ Regulatory T cell function. J Immunol. (2021) 206:1776–83. doi: 10.4049/jimmunol.2000051 PMC8026672 33789982

[47] Kadono T Venturi GM Steeber DA Tedder TF. Leukocyte rolling velocities and migration are optimized by cooperative L-selectin and intercellular adhesion molecule-1 functions. J Immunol. (2002) 169:4542–50. doi: 10.4049/jimmunol.169.8.4542 12370391

[48] Wagers AJ Lowe JB Kansas GS. An important role for the alpha 1,3 fucosyltransferase, FucT-VII, in leukocyte adhesion to E-selectin. Blood. (1996) 88:2125–32. doi: 10.1182/blood.V88.6.2125.bloodjournal8862125 8822932

[49] Katayama T Ikeda Y Handa M Tamatani T Sakamoto S Ito M. Immunoneutralization of glycoprotein ibα Attenuates endotoxin-induced interactions of platelets and leukocytes with rat venular endothelium *in vivo* . Circ Res. (2000) 86:1031–7. doi: 10.1161/01.res.86.10.1031 10827132

[50] Li X Tu L Murphy PG Kadono T Steeber DA Tedder TF. CHST1 and CHST2 sulfotransferase expression by vascular endothelial cells regulates shear-resistant leukocyte rolling via L-selectin. J Leukoc Biol. (2001) 69:565–74. doi: 10.1189/jlb.69.4.565 11310842

[51] Souto F Alves-Filho J Freitas A Spiller F Martins M Basile-Filho A. CCR2 expression on neutrophils leads to detrimental tissue infiltration during sepsis. Crit Care. (2009) 13:P9. doi: 10.1186/cc7811

[52] Tahvanainen J Kallonen T Lähteenmäki H Heiskanen KM Westermarck J Rao KVS. PRELI is a mitochondrial regulator of human primary T-helper cell apoptosis, STAT6, and Th2-cell differentiation. Blood. (2009) 113:1268–77. doi: 10.1182/blood-2008-07-166553 18945965

[53] Kaminker JD Timoshenko AV. Expression, regulation, and functions of the galectin-16 gene in human cells and tissues. Biomolecules. (2021) 11:1909. doi: 10.3390/biom11121909 34944551 PMC8699332

[54] Shen YH Zhang L Ren P Nguyen MT Zou S Wu D. AKT2 confers protection against aortic aneurysms and dissections. Circ Res. (2013) 112:618–32. doi: 10.1161/circresaha.112.300735 PMC3586338 23250987

[55] Luo Z Fujio Y Kureishi Y Rudic RD Daumerie G Fulton D. Acute modulation of endothelial Akt/PKB activity alters nitric oxide–dependent vasomotor activity *in vivo* . J Clin Investig. (2000) 106:493–9. doi: 10.1172/jci9419 PMC380252 10953024

[56] Stabile E Zhou YF Saji M Castagna M Shou M Kinnaird TD. Akt controls vascular smooth muscle cell proliferation *in vitro* and *in vivo* by delaying G1/S exit. Circ Res. (2003) 93:1059–65. doi: 10.1161/01.res.0000105086.31909.1b 14605018

[57] Kataoka H Kume N Miyamoto S Minami M Morimoto M Hayashida K. Oxidized LDL modulates bax/bcl-2 through the lectinlike ox-LDL receptor-1 in vascular smooth muscle cells. Arter Thromb Vasc Biol. (2001) 21:955–60. doi: 10.1161/01.atv.21.6.955 11397703

[58] Zaitoun IS Wintheiser CM Jamali N Wang S Suscha A Darjatmoko SR. Bcl-2 expression in pericytes and astrocytes impacts vascular development and homeostasis. Sci Rep. (2019) 9:9700. doi: 10.1038/s41598-019-45915-4 31273232 PMC6609701

[59] Brophy CM Knoepp L Xin J Pollock JS. Functional expression of NOS 1 in vascular smooth muscle. Am J Physiol-Hear Circ Physiol. (2000) 278:H991–7. doi: 10.1152/ajpheart.2000.278.3.h991 10710369

[60] Julian L Olson MF. Rho-associated coiled-coil containing kinases (ROCK). Small GTPases. (2014) 5:e29846. doi: 10.4161/sgtp.29846 25010901 PMC4114931

[61] Molla MR Shimizu A Komeno M Rahman NIA Soh JEC Nguyen LKC. Vascular smooth muscle RhoA counteracts abdominal aortic aneurysm formation by modulating MAP4K4 activity. Commun Biol. (2022) 5:1071. doi: 10.1038/s42003-022-04042-z 36207400 PMC9546906

[62] Shigematsu K Koyama H Olson NE Cho A Reidy MA. Phosphatidylinositol 3-kinase signaling is important for smooth muscle cell replication after arterial injury. Arter Thromb Vasc Biol. (2000) 20:2373–8. doi: 10.1161/01.atv.20.11.2373 11073840

[63] Singh S Brocker C Koppaka V Chen Y Jackson BC Matsumoto A. Aldehyde dehydrogenases in cellular responses to oxidative/electrophilicstress. Free Radic Biol Med. (2013) 56:89–101. doi: 10.1016/j.freeradbiomed.2012.11.010 23195683 PMC3631350

[64] Pan X Grigoryeva L Seyrantepe V Peng J Kollmann K Tremblay J. Serine carboxypeptidase SCPEP1 and cathepsin A play complementary roles in regulation of vasoconstriction via inactivation of endothelin-1. PloS Genet. (2014) 10:e1004146. doi: 10.1371/journal.pgen.1004146 24586188 PMC3937211

[65] Kokkoris S Andrews P Webb DJ. Role of calcitonin gene-related peptide in cerebral vasospasm, and as a therapeutic approach to subarachnoid hemorrhage. Front Endocrinol. (2012) 3:135. doi: 10.3389/fendo.2012.00135 PMC3498620 23162536

[66] Russell FA King R Smillie S-J Kodji X Brain SD. Calcitonin gene-related peptide: physiology and pathophysiology. Physiol Rev. (2014) 94:1099–142. doi: 10.1152/physrev.00034.2013 PMC4187032 25287861

[67] Rajesh M Mukhopadhyay P Haskó G Pacher P. Cannabinoid CB1 receptor inhibition decreases vascular smooth muscle migration and proliferation. Biochem Biophys Res Commun. (2008) 377:1248–52. doi: 10.1016/j.bbrc.2008.10.159 PMC2646252 18996082

[68] Doukas P Hartmann O Frankort J Arlt B Krabbe H Jacobs MJ. Postoperative bioactive adrenomedullin is associated with the onset of ARDS and adverse outcomes in patients undergoing open thoracoabdominal aortic surgery. Sci Rep. (2024) 14:12795. doi: 10.1038/s41598-024-63412-1 38834580 PMC11150250

[69] Passaglia P Gonzaga NA Tirapelli DPC Tirapelli LF Tirapelli CR. Pharmacological characterisation of the mechanisms underlying the relaxant effect of adrenomedullin in the rat carotid artery. J Pharm Pharmacol. (2014) 66:1734–46. doi: 10.1111/jphp.12299 25117796

[70] Márquez M Muñoz M Córdova A Puebla M Figueroa XF. Connexin 40-mediated regulation of systemic circulation and arterial blood pressure. J Vasc Res. (2023) 60:87–100. doi: 10.1159/000531035 37331352

[71] Shoja MM Tubbs RS Ansarin K. The role of myoendothelial gap junctions in the formation of arterial aneurysms: The hypothesis of “connexin 43:40 stoichiometry. Méd Hypotheses. (2007) 69:575–9. doi: 10.1016/j.mehy.2007.01.035 17374558

[72] Förstermann U Sessa WC. Nitric oxide synthases: regulation and function. Eur Hear J. (2012) 33:829–37. doi: 10.1093/eurheartj/ehr304 PMC3345541 21890489

[73] Fu Y Liu H Li K Wei P Alam N Deng J. C-reactive protein deficiency ameliorates experimental abdominal aortic aneurysms. Front Immunol. (2023) 14:1233807. doi: 10.3389/fimmu.2023.1233807 37753091 PMC10518468

[74] Xin H He X Li J Guan X Liu X Wang Y. Profiling of the full-length transcriptome in abdominal aortic aneurysm using nanopore-based direct RNA sequencing. Open Biol. (2022) 12:210172. doi: 10.1098/rsob.210172 35104432 PMC8807055

[75] Rizo-Téllez SA Sekheri M Filep JG. C-reactive protein: a target for therapy to reduce inflammation. Front Immunol. (2023) 14:1237729. doi: 10.3389/fimmu.2023.1237729 37564640 PMC10410079

[76] Thiele JR Habersberger J Braig D Schmidt Y Goerendt K Maurer V. Dissociation of pentameric to monomeric C-reactive protein localizes and aggravates inflammation. Circulation. (2014) 130:35–50. doi: 10.1161/circulationaha.113.007124 24982116

[77] Hui DY. A no-no for nonO and JNK in extracellular matrix homeostasis and vascular stability. Arter Thromb Vasc Biol. (2007) 27:1677–8. doi: 10.1161/atvbaha.107.146894 17634520

[78] Patel R Hall S Lanford H Ward N Grespin RT Figueroa M. Signaling through the IL-6-STAT3 pathway promotes proteolytically-active macrophage accumulation necessary for development of small AAA. Vasc Endovasc Surg. (2023) 57:433–44. doi: 10.1177/15385744231152961 PMC10238619 36639147

[79] Yang B Wang X Ying C Peng F Xu M Chen F. Long Noncoding RNA SNHG16 Facilitates Abdominal Aortic Aneurysm Progression through the miR-106b-5p/STAT3 Feedback Loop. J Atheroscler Thromb. (2021) 28:66–78. doi: 10.5551/jat.52274 32612026 PMC7875146

[80] Chen J Liao M Gao X Zhong Q Tang T Yu X. IL-17A induces pro-inflammatory cytokines production in macrophages via MAPKinases, NF-κB and AP-1. Cell Physiol Biochem. (2013) 32:1265–74. doi: 10.1159/000354525 24247374

[81] Erbel C Akhavanpoor M Okuyucu D Wangler S Dietz A Zhao L. IL-17A influences essential functions of the monocyte/macrophage lineage and is involved in advanced murine and human atherosclerosis. J Immunol. (2014) 193:4344–55. doi: 10.4049/jimmunol.1400181 PMC4201987 25261478

[82] Wu Q Cheng Z Zhou Y Zhao Y Li J Zhou X. A novel STAT3 inhibitor attenuates angiotensin II-induced abdominal aortic aneurysm progression in mice through modulating vascular inflammation and autophagy. Cell Death Dis. (2020) 11:131. doi: 10.1038/s41419-020-2326-2 32071300 PMC7028955

[83] Fu W Liu H Wei P Xia C Yu Q Tian K. Genetic deficiency of protein inhibitor of activated STAT3 suppresses experimental abdominal aortic aneurysms. Front Cardiovasc Med. (2023) 10:1092555. doi: 10.3389/fcvm.2023.1092555 37008329 PMC10050368

[84] Huang B Lang X Li X. The role of IL-6/JAK2/STAT3 signaling pathway in cancers. Front Oncol. (2022) 12:1023177. doi: 10.3389/fonc.2022.1023177 36591515 PMC9800921

[85] Zhang D Sun M Samols D Kushner I. STAT3 participates in transcriptional activation of the C-reactive protein gene by interleukin-6 (∗). J Biol Chem. (1996) 271:9503–9. doi: 10.1074/jbc.271.16.9503 8621622

[86] Ngwa DN Pathak A Agrawal A. IL-6 regulates induction of C-reactive protein gene expression by activating STAT3 isoforms. Mol Immunol. (2022) 146:50–6. doi: 10.1016/j.molimm.2022.04.003 PMC9811655 35430542

[87] Wohlfahrt T Rauber S Uebe S Luber M Soare A Ekici A. PU.1 controls fibroblast polarization and tissue fibrosis. Nature. (2019) 566:344–9. doi: 10.1038/s41586-019-0896-x PMC6526281 30700907

[88] Devaraj S Jialal I. C-reactive protein polarizes human macrophages to an M1 phenotype and inhibits transformation to the M2 phenotype. Arter Thromb Vasc Biol. (2011) 31:1397–402. doi: 10.1161/atvbaha.111.225508 PMC3099471 21415385

[89] Trial J Potempa LA Entman ML. The role of C-reactive protein in innate and acquired inflammation: new perspectives. Inflammation Cell Signal. (2016) 3(2):e1409. PMC5058362 27738646

[90] Li D Li J Liu H Zhai L Hu W Xia N. Pathogenic Tconvs promote inflammatory macrophage polarization through GM-CSF and exacerbate abdominal aortic aneurysm formation. FASEB J. (2022) 36:e22172. doi: 10.1096/fj.202101576r 35133017 PMC9303938

[91] Guo Q Jin Y Chen X Ye X Shen X Lin M. NF-κB in biology and targeted therapy: new insights and translational implications. Signal Transduct Target Ther. (2024) 9:53. doi: 10.1038/s41392-024-01757-9 38433280 PMC10910037

[92] Trigueros-Motos L González-Granado JM Cheung C Fernández P Sánchez-Cabo F Dopazo A. Embryological-origin–dependent differences in homeobox expression in adult aorta. Arter Thromb Vasc Biol. (2013) 33:1248–56. doi: 10.1161/atvbaha.112.300539 23448971

[93] Alfaddagh A Martin SS Leucker TM Michos ED Blaha MJ Lowenstein CJ. Inflammation and cardiovascular disease: From mechanisms to therapeutics. Am J Prev Cardiol. (2020) 4:100130. doi: 10.1016/j.ajpc.2020.100130 34327481 PMC8315628

[94] Ghosh A DiMusto PD Ehrlichman LK Sadiq O McEvoy B Futchko JS. The role of extracellular signal-related kinase during abdominal aortic aneurysm formation. J Am Coll Surg. (2012) 215:668–680.e1. doi: 10.1016/j.jamcollsurg.2012.06.414 22917644 PMC3586428

[95] Yoshimura K Aoki H Ikeda Y Fujii K Akiyama N Furutani A. Regression of abdominal aortic aneurysm by inhibition of c-Jun N-terminal kinase. Nat Med. (2005) 11:1330–8. doi: 10.1038/nm1335 16311603

[96] Kajimoto K Miyauchi K Kasai T Shimada K Kojima Y Shimada A. Short-term 20-mg atorvastatin therapy reduces key inflammatory factors including c-Jun N-terminal kinase and dendritic cells and matrix metalloproteinase expression in human abdominal aortic aneurysmal wall. Atherosclerosis. (2009) 206:505–11. doi: 10.1016/j.atherosclerosis.2009.03.028 19406402

[97] Aoki H Yoshimura K Matsuzaki M. Turning back the clock: regression of abdominal aortic aneurysms via pharmacotherapy. J Mol Med. (2007) 85:1077–88. doi: 10.1007/s00109-007-0213-2 17522832

[98] Dong Q Wright JR. Expression of C-reactive protein by alveolar macrophages. J Immunol (Baltim Md: 1950). (1996) 156:4815–20. doi: 10.4049/jimmunol.156.12.4815 8648129

[99] Kaplan M Shur A Tendler Y. M1 Macrophages but Not M2 Macrophages Are Characterized by Upregulation of CRP Expression via Activation of NFκB: a Possible Role for Ox-LDL in Macrophage Polarization. Inflammation. (2018) 41:1477–87. doi: 10.1007/s10753-018-0793-8 29687414

[100] Xu C Zarins CK Glagov S. Aneurysmal and occlusive atherosclerosis of the human abdominal aorta. J Vasc Surg. (2001) 33:91–6. doi: 10.1067/mva.2001.109744 11137928

[101] Zankl AR Schumacher H Krumsdorf U Katus HA Jahn L Tiefenbacher CP. Pathology, natural history and treatment of abdominal aortic aneurysms. Clin Res Cardiol. (2007) 96:140–51. doi: 10.1007/s00392-007-0472-5 17180573

[102] Yuan Z Lu Y Wei J Wu J Yang J Cai Z. Abdominal aortic aneurysm: roles of inflammatory cells. Front Immunol. (2021) 11:609161. doi: 10.3389/fimmu.2020.609161 33613530 PMC7886696

[103] Brady AR Thompson SG Fowkes FGR Greenhalgh RM Powell JT Participants USAT. Abdominal aortic aneurysm expansion. Circulation. (2004) 110:16–21. doi: 10.1161/01.cir.0000133279.07468.9f 15210603

[104] Le S Wu J Liu H Du Y Wang D Luo J. Single-cell RNA sequencing identifies interferon-inducible monocytes/macrophages as a cellular target for mitigating the progression of abdominal aortic aneurysm and rupture risk. Cardiovasc Res. (2024) 120:1351–64. doi: 10.1093/cvr/cvae117 38836630

[105] Zhang K Yue J Yin L Chen J Chen Y Hu L. Comprehensive bioinformatics analysis revealed potential key genes and pathways underlying abdominal aortic aneurysm. Comput Struct Biotechnol J. (2023) 21:5423–33. doi: 10.1016/j.csbj.2023.10.052 PMC10665597 38022704

[106] Yuan Z Shu L Fu J Yang P Wang Y Sun J. Single-cell RNA sequencing deconstructs the distribution of immune cells within abdominal aortic aneurysms in mice. Arter Thromb Vasc Biol. (2024) 44:1986–2003. doi: 10.1161/atvbaha.124.321129 39051127

[107] Xiong J Chen G Lin B Zhong L Jiang X Lu H. Integrative analysis of single-Cell RNA sequencing and experimental validation in the study of abdominal aortic aneurysm progression. Gene. (2024) 929:148820. doi: 10.1016/j.gene.2024.148820 39103059

[108] Bergwerff M Verberne ME DeRuiter MC Poelmann RE Gittenberger-de-Groot AC. Neural crest cell contribution to the developing circulatory system. Circ Res. (1998) 82:221–31. doi: 10.1161/01.res.82.2.221 9468193

[109] Pfaltzgraff ER Shelton EL Galindo CL Nelms BL Hooper CW Poole SD. Embryonic domains of the aorta derived from diverse origins exhibit distinct properties that converge into a common phenotype in the adult. J Mol Cell Cardiol. (2014) 69:88–96. doi: 10.1016/j.yjmcc.2014.01.016 24508561 PMC4034360

[110] Oh SJ Kim EN Kim CJ Choi J-S Kim K-B. The effect of C-reactive protein deposition on myocardium with ischaemia–reperfusion injury in rats. Interact Cardiov Th. (2017) 25:260–7. doi: 10.1093/icvts/ivx107 28475685

[111] Kim EN Kim CJ Kim SR Song J-A Choe H Kim K-B. High serum CRP influences myocardial miRNA profiles in ischemia-reperfusion injury of rat heart. PloS One. (2019) 14:e0216610. doi: 10.1371/journal.pone.0216610 31063484 PMC6504103

